# A source of isotopically light organic carbon in a low-pH anoxic marine zone

**DOI:** 10.1038/s41467-021-21871-4

**Published:** 2021-03-11

**Authors:** Cristian A. Vargas, Sebastian I. Cantarero, Julio Sepúlveda, Alexander Galán, Ricardo De Pol-Holz, Brett Walker, Wolfgang Schneider, Laura Farías, Marcela Cornejo D’Ottone, Jennifer Walker, Xiaomei Xu, Joe Salisbury

**Affiliations:** 1 Department of Aquatic System, Faculty of Environmental Sciences & Environmental Sciences Center EULA Chile, Universidad de Concepción, Concepción, Chile; 2 Millennium Institute of Oceanography (IMO), Universidad de Concepción, Concepción, Chile; 3 Coastal Social-Ecological Millennium Institute (SECOS), Universidad de Concepción, Concepción, Chile; 4 Department of Geological Sciences and Institute of Arctic and Alpine Research (INSTAAR), University of Colorado Boulder, Boulder, CO USA; 5 Centro de Investigación de Estudios Avanzados del Maule (CIEAM), Departamento de Obras Civiles, Facultad de Ciencias de la Ingeniería, Universidad Católica del Maule, Talca, Chile; 6 Centro Regional de Estudios Ambientales (CREA), Universidad Católica de la Santísima Concepción, Concepción, Chile; 7 Centro de Investigación GAIA-Antártica (CIGA) and Network for Extreme Environment Research (NEXER), Universidad de Magallanes, Punta Arenas, Chile; 8 Department of Earth and Environmental Sciences, University of Ottawa, Ottawa, Canada; 9 Department of Oceanography, Universidad de Concepcion, Concepcion, Chile; 10 Center for Climate and Resilience Research (CR)2, Santiago, Chile; 11 Escuela de Ciencias del Mar, P. Universidad Católica de Valparaíso, Valparaíso, Chile; 12 Department of Earth System Science, University of California, Irvine, CA USA; 13 Ocean Process Analysis Lab, University of New Hampshire, Durham, NC USA

**Keywords:** Biochemistry, Biogeochemistry, Ocean sciences, Chemistry

## Abstract

Geochemical and stable isotope measurements in the anoxic marine zone (AMZ) off northern Chile during periods of contrasting oceanographic conditions indicate that microbial processes mediating sulfur and nitrogen cycling exert a significant control on the carbonate chemistry (pH, A_T_, DIC and *p*CO_2_) of this region. Here we show that in 2015, a large isotopic fractionation between DIC and POC, a DIC and N deficit in AMZ waters indicate the predominance of in situ dark carbon fixation by sulfur-driven autotrophic denitrification in addition to anammox. In 2018, however, the fractionation between DIC and POC was significantly lower, while the total alkalinity increased in the low-pH AMZ core, suggesting a predominance of heterotrophic processes. An isotope mass-balance model demonstrates that variations in the rates of sulfur- and nitrogen-mediated carbon fixation in AMZ waters contribute ~7–35% of the POC exported to deeper waters. Thus, dark carbon fixation should be included in assessments of future changes in carbon cycling and carbonate chemistry due to AMZ expansion.

## Abstract (web summary)

Anoxic marine zones are expanding and intensifying with climate change. Here the authors show that microbial dark carbon fixation influences the carbonate system and the stable isotope composition in waters off Chile, contributing up to 35% of the organic carbon reaching the mesopelagic region.

## Introduction

Dissolved oxygen plays a key role in shaping the structure of marine ecosystems, the spatial and temporal distribution of marine organisms^1^, and regulates both metabolic and biogeochemical processes^2^. Since oxygen (O_2_) is slowly depleted in the ocean with depth, in some regions this leads to the formation of steady-state O_2_-deficient intermediate layers commonly known as oxygen minimum zones (OMZs)^3,4^. O_2_ varies geographically among OMZs, but it generally falls below <20 μmol kg^−1^ ^5^. Marine time series indicate a substantial shoaling of the upper OMZ boundary over the past decades along several eastern boundary-current systems, such as the Subarctic Pacific^6^, the eastern equatorial region^7^, and the northeast Pacific Ocean^8^. Notably, newly developed oxygen sensors^9^ have demonstrated that O_2_ can fall below sensor-specific detection limits (~3 nmol L^−1^) within a significant fraction of OMZ waters.

Due to their extreme oxygen-depletion, OMZs like the one along the eastern tropical South Pacific (ETSP) coast off Peru and Chile have been recently redefined as anoxic marine zones (AMZs)^10^. AMZs are often distinguished from OMZs by the accumulation of nitrite, which typically occurs when O_2_ falls below the nanomolar detection limit^10^. In these anoxic zones, nitrate- and sulfate-reducing bacteria, as well as methanotrophic and canonical denitrifying bacteria dominate heterotrophic processes^10,11^. The combination of microbially mediated remineralization process, which consumes O_2_ and produces dissolved inorganic carbon (DIC), in addition to weak ventilation, leads to the establishment and persistence of O_2_-depleted waters. Organic carbon oxidation in AMZ waters is driven by microorganisms able to utilize alternative electron acceptors, which results in excess CO_2_ and lower pH (< 7.8)^12^. Additionally, under such O_2_-limited conditions, chemolithoautotrophic processes that consume DIC are dominated by bacterial sulfur-driven autotrophic denitrification (SDAD) and anaerobic ammonium oxidation (Anammox), although oxygen-dependent nitrifying microorganisms (i.e., ammonium- and nitrite-oxidizers) also operate, but at lower rates^13,14^. In consequence, heterotrophic and chemoautotrophic processes associated with AMZs influence marine carbon dynamics and the cycling of key elements for marine productivity, but also the carbonate chemistry (pH, DIC, and *p*CO_2_). The latter has been scarcely studied in these regions. For instance, heterotrophic activity reduces the efficiency of the biological carbon pump by consuming organic matter and releasing CO_2_, whereas chemoautotrophic processes can increase the efficiency of this pumping by fixing additional CO_2_ and generating new organic carbon in intermediate waters^12,15^.

Recent work has shown that the nitrite-rich AMZ in the ETSP off northern Chile is an extreme end-member within the spectrum of AMZ ecosystems, where O_2_ is mostly absent, except during sporadic intrusions^10^. Gene surveys in this region have shown that significant fractions of picoplankton in AMZ waters are chemoautotrophs associated with the cycling of sulfur and nitrogen, such as SDAD and anammox^16,17^. Furthermore, recent studies using isotope-mixing models in other AMZs also suggest that dark carbon fixation could contribute significantly to sinking carbon fluxes^18^. However, the influence of these chemoautotrophic processes on DIC and *p*CO_2_ levels in AMZ waters remains poorly understood. Since the biomass from chemoautotrophs inhabiting OMZs and AMZs is characterized by low δ^13^C signatures, which is modulated by both the uptake of remineralised and ^13^C-depleted CO_2_^19^, and the use of carbon fixation pathways such as the acetyl coenzyme A^20^, exported organic matter from these regions should preserve this isotopically depleted signature.

In view of the projected increases in both the extension and intensity of oxygen-deficient zones^7,8^, it remains crucial to improve our understanding about the natural variability in the carbonate chemistry system of AMZs to better predict future changes. Models suggest that a doubling of surface water *p*CO_2_ could lead to a doubling or more of *p*CO_2_ in OMZ/AMZ waters^21^, mostly due to the reduction of seawater´s buffer capacity by the acidic components of the normal Redfield cycle^22^. However, these projections ignore the impact of microbially driven processes linked to sulfur and/or nitrogen on biogeochemical cycles, including carbon cycling. Moreover, modeling studies based on the oceanic uptake of anthropogenic CO_2_ indicate that surface waters in eastern boundary systems, such as the Humboldt Current, are likely to become more corrosive to CaCO_3_^23^. Nevertheless, the extent to which O_2_ concentrations, the sulfur and nitrogen cycles, and the carbonate system are linked in AMZs remains poorly understood^12,15^.

Here, by using a suite of physical (i.e. temperature, salinity) and chemical parameters (i.e., oxygen, inorganic nutrients, pH, total alkalinity, carbon pool concentration, and their stable isotopic composition), we hypothesize that the unique microbial communities inhabiting the AMZ ecosystem of the ETSP off northern Chile^10,17^ constitute an important modulator of the carbonate chemistry variability in this region. These results are then incorporated into a simple carbon isotope box model to determine potential sources of organic matter and calculate the contribution of microbial autotrophic production in AMZ waters to organic carbon export to the mesopelagic region.

## Results

### Physical oceanographic conditions

**Fig. 1:**
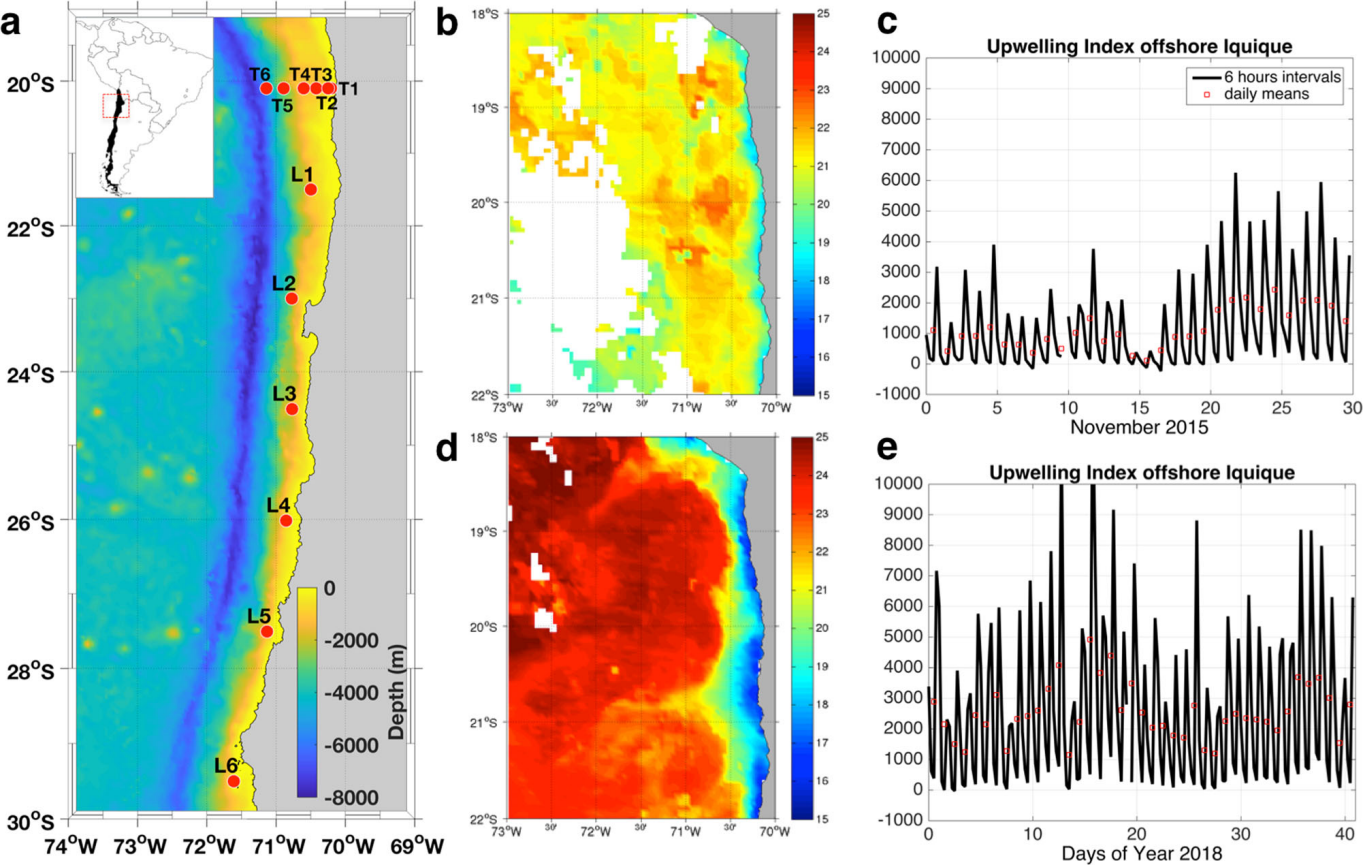
Sampling stations and oceanographic conditions during the expeditions. **a** Study area, including six sampling stations (T1–T6), and a longitudinal section from 20° down to 29.5°S, including six sampling stations (L1–L6). **b** 25 November–2 December 2015 composite of MODIS-Aqua SST for LowPhox I; units are in °C; persisting clouds appear in white. **c** Upwelling Indices for the oceanic region offshore Iquique for November 2015; black: 6-h intervals and red daily averages; units are in kg m^−1^ s^−1^. **d** 2–9 February 2018 composite of MODIS-Agua SST for LowPhox II; units are in °C; persisting clouds appear in white. **e** Upwelling index for the oceanic region offshore Iquique from 1 January to 12 February 2018; the black lines are 6-h intervals and red dots correspond to daily averages; units are in kg m^−1^ s^−1^.

During the second half of November 2015 (austral spring; early upwelling season), coastal upwelling started its seasonal intensification, as reflected in an upwelling index of ~2000 kg m^−1^ s^−1^ off the coast of Iquique (Fig. 1c). Sea surface temperature was ~21–22 °C, with a narrow upwelling signal < 19 °C close to the coast (Fig. 1b). During early February 2018 (austral summer; upwelling peak) the upwelling index was ~33% stronger (3000 kg m^−1^ s^−1^, Fig. 1e), whereas sea surface temperatures were as low as 17 °C prevailed along the coast of northern Chile, including our coastal stations T1 and T2. These intense upwelling conditions were reflected in high surface chlorophyll a concentrations of about 20 mg m^−3^ within 50 km from the coastline, compared to lower concentrations of 5 mg m^−3^ in 2015 (Supplementary Fig. 1a, b). Another difference between the 2015 and 2018 cruises was the hydrographic properties of the upper thermocline. Unlike 2015, the water column in 2018 was characterized by the intrusion of well ventilated, low salinity water subducted off central Chile (Supplementary Fig. 1c)^24^. This water mass has a mean temperature of 12 °C and intrudes the upper pycnocline debilitating the AMZ, feeding the upwelling process during 2018. Anomalies of sea surface temperature for November and December 2015 did not indicate that the El Niño 2015/16 had yet arrived at the study area.

### Carbon chemistry and C:N:P stoichiometry in the AMZ

**Fig. 2:**
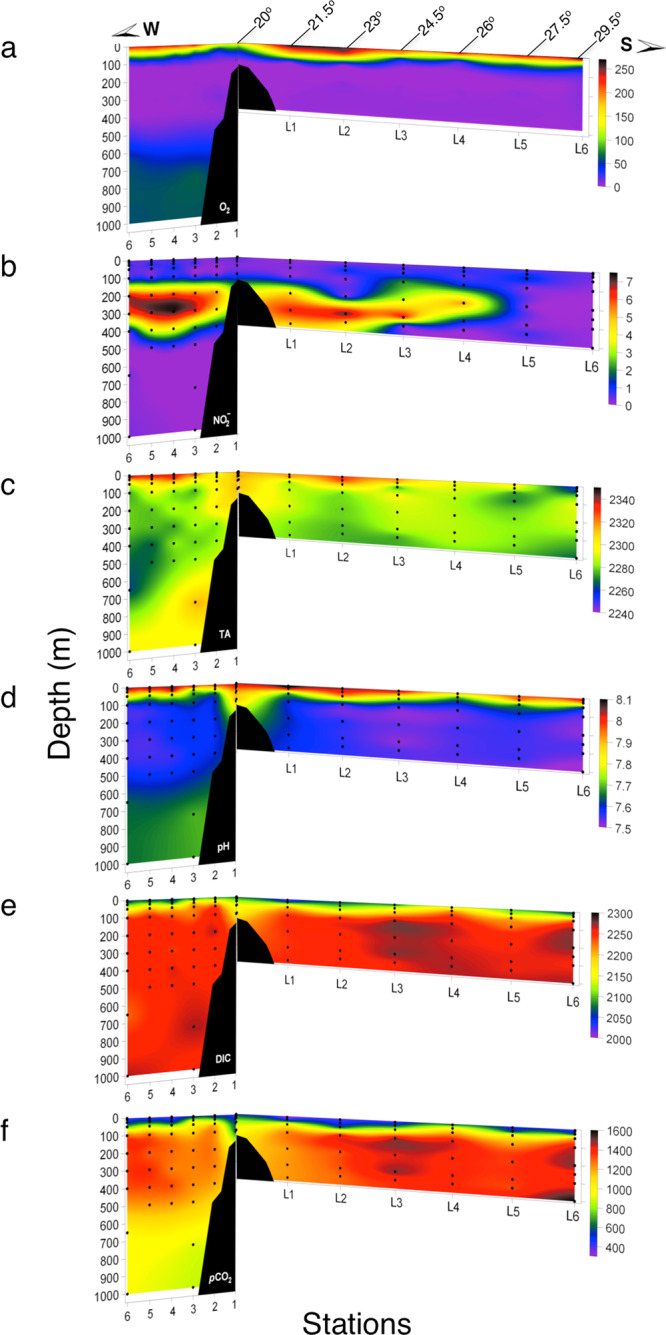
Spatial variability in oxygen, nitrate, and carbonate chemistry during 2015. Vertical sections of (**a**) dissolved oxygen (μmol kg^−1^), (**b**) nitrite (μmol kg^−1^), (**c**) total alkalinity (μmol kg^−1^), (**d**) pH_T_, (**e**), dissolved inorganic carbon (μmol kg^−1^), and *p*CO_2_ (μatm) during the research cruise in 2015. The sections included both a cross-shelf section at 20°S (1–6), and a latitudinal section along the coast (L1–L6). The black dots represent sample locations.

During 2015, oxygen-deficient conditions (<0.02 μmol kg^−1^) were observed between steep upper (~80–100 m) and lower (~400–450 m) oxyclines along the coast off northern Chile (20.0°–29.5 °S), as well as perpendicular to the coast across a neritic-pelagic transect off Iquique (20 °S; Fig. 2a). A nitrite maximum (NO_2_^−^ > 5 μmol kg^−1^ and up to 7.5 μmol kg^−1^), characteristic of anoxic conditions, was detected in the core of the AMZ between 200 and 300 m depth along the coast from ca. 20 °S to 26 °S (Station L1–L4, Fig. 1b), as well as offshore from stations T4 to T6 (Fig. 2b). Below the AMZ core, O_2_ concentrations increased from under detection limit up to 80 μmol kg^−1^ (Fig. 2a). Deficient O_2_ waters were also characterized by low pH (~7.5–7.6) and high *p*CO_2_ (~1200–1600 μatm) (Fig. 2d–f) compared to surface oxygenated waters (pH 7.8–8.1 and *p*CO_2_ 400–700 μatm). In the AMZ core, the DIC and *p*CO_2_ were lower, whereas pH was comparatively higher in the AMZ core around the NO_2_^−^ maximum, compared to surrounding anoxic waters (e.g. see Stn L6, Fig. 2d, e).

**Fig. 3:**
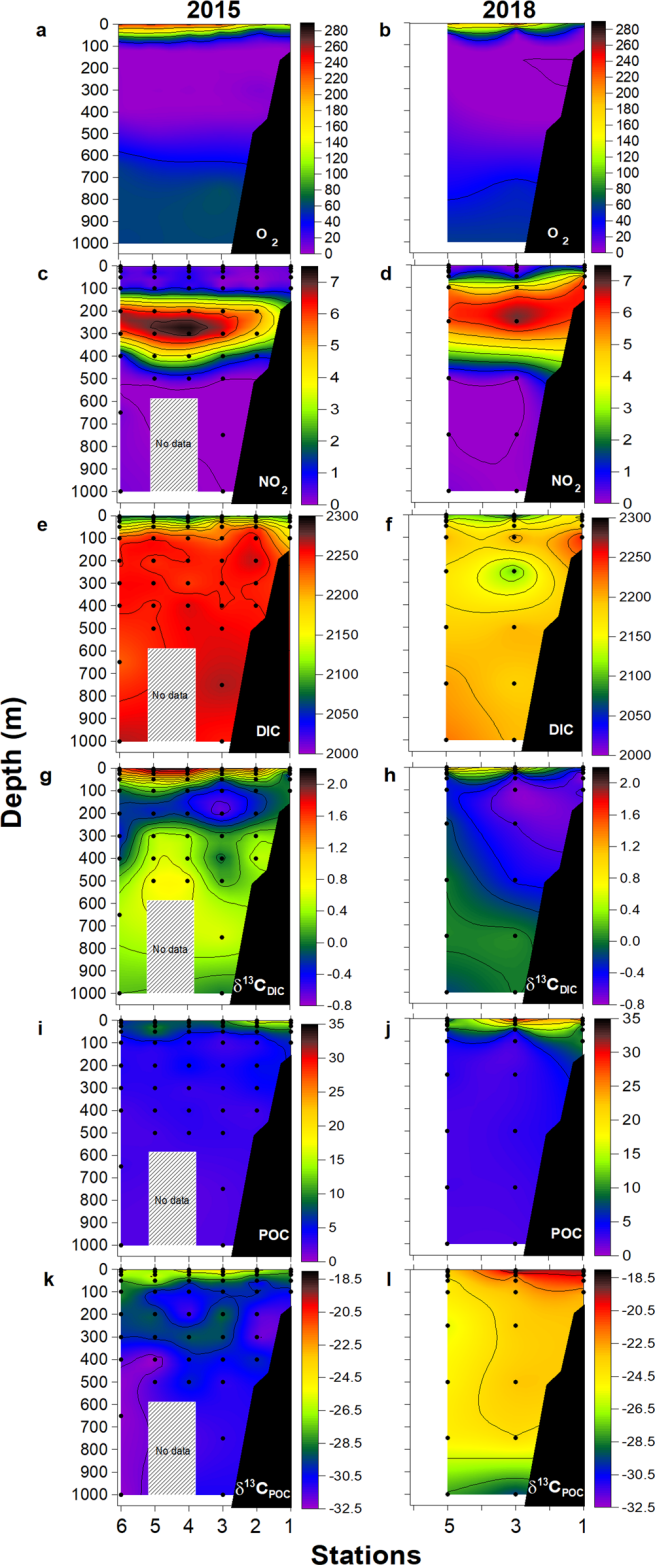
Comparative conditions in 2015 vs. 2018 in a cross-shelf section off Iquique (20 °S). Vertical cross-shelf sections of oxygen, nitrite, dissolved inorganic carbon (DIC) and particulate organic carbon (POC) pools and their ^13^C isotopic values during research cruises in 2015 and 2018. (**a**, **b**) Dissolved oxygen (μmol kg^−1^), (**c**, **d**) nitrite (μmol kg^−1^), (**e**, **f**) dissolved inorganic carbon (μmol kg^−1^), (**g**, **h**) δ^13^C_DIC_ (‰), (**i**, **j**) particulate organic carbon (μmol kg^−1^), and (**k**, **l**) δ^13^C_POC_ (‰). The black dots represent sample locations. Gray box represents area with non-collected data.

**Fig. 4:**
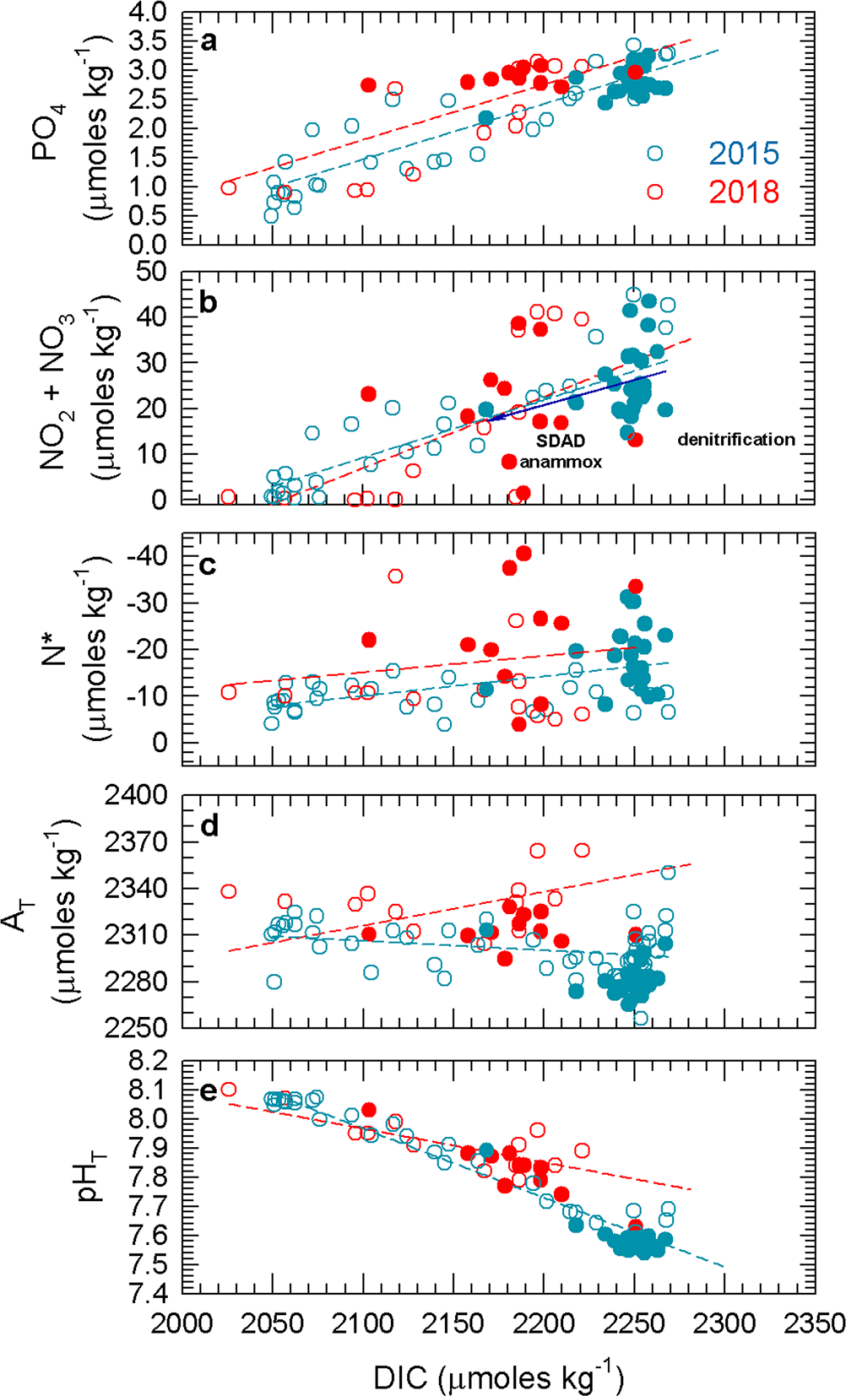
Relationship between dissolved inorganic carbon (DIC) versus nutrients and other carbonate chemistry parameters. (**a**) PO_4_^3−^, (**b**) inorganic nitrogen (NO_2_^−^ + NO_3_^−^), (**c**) N*, (**d**) total pH (pH_T_), and (**e**) total alkalinity (A_T_), within the range from surface to 1000 m water depth during 2015 and 2018 expeditions. Filled symbols correspond to data points associated with O_2_ concentrations < 20 μmol kg^−1^. The correlations observed in low oxygen waters (filled points) for DIC versus nitrogen at 2015 is given and indicated by the dark blue line (**b**). Labels in (**b**) were included to emphasize the processes highlighted in the stoichiometric analysis (e.g. Annamox, sulfur-driven autotrophic denitrification (SDAD), and denitrification).

A comparative analysis of seawater chemistry between 2015 and 2018 in the cross-shelf transect off Iquique (20 °S; Stations T1–T6), evidenced highly contrasting conditions (Fig. 3). Whereas in 2015 the AMZ core (ca. 7.5 μmol NO_2_^−^ kg^−1^) was deeper and located farther offshore, in 2018 the NO_2_^−^ maximum (<6.5 μmol kg^−1^) was slightly shallower and closer to the coast around Stn T3, whereas the slope of both O_2_ and NO_2_^−^ isolines suggest the shoaling of low-oxygen waters over the continental shelf in 2018 (Fig. 3a–b, c–d). High DIC concentrations were observed in subsurface waters during 2015 (>2250 μmol kg^−1^; Fig. 3e), whereas in 2018 high DIC values were restricted to the continental shelf at Stn 1 (Fig. 3f). The relationship between DIC and PO_4_^3−^ concentration was relatively similar between both years as evidenced by similar slopes (Fig. 4a). However, during 2015, some regions of the oxygen-depleted waters showed low inorganic N content (i.e., NO_3_^−^ + NO_2_^−^, ~20 μmol kg^−1^) and a high N deficiency (N*; −10 to −20 μmol kg^−1^), associated with relatively lower DIC concentration (2160–2210 μmol kg^−1^) (Fig. 4b, c). As we discuss in more detail below, high N* (>−10 µmol kg^−1^) associated with increasing DIC (~2250 µmol kg^−1^) in low-O_2_ waters (filled blue dots in Fig. 4) appears to be related to denitrification; on the other hand, high N* values at lower DIC concentration (<2210 µmol kg^−1^) in suboxic/anoxic conditions could be associated with both SDAD and anammox processes (Fig. 4b, c). In fact, a decrease in DIC of up to 30 μmol in the AMZ core suggests the prevalence of dark carbon fixation over heterotrophic denitrification in the DIC pool of some stations sampled during 2015 (Fig. 4b). Moreover, since both SDAD and anammox consume H^+^, the high A_T_ (2310 µmol kg^−1^), which is likely related to the consumption of nitrate and nitrite, results in the more elevated pH (~7.9) observed in oxygen-depleted waters (Stn 5) (Fig. 4d, e). Conversely, in 2018, we observed decreasing inorganic N and high N*, but with lower DIC concentrations and high A_T_ in comparison to 2015 (filled red dots in Fig. 4b–d). Higher DIC concentration in the AMZ during 2015 resulted in lower pH_T_ compared to 2018 (7.5–7.6 vs. 7.8–7.9, respectively; Fig. 4e). Indeed, the concentration of aqueous CO_2_ (CO_2-aq_; Supplementary Fig. 2a) in the AMZ core (i.e., NO_2_^−^ maximum) was significantly higher in 2015 (~50, and up to ~60 μmol kg^−1^) than in 2018 (~15, and up to 45 μmol kg^−1^). The relationship between O_2_ and CO_2-aq_ in 2015 showed a large range of CO_2-aq_ (25–60 μmol kg^−1^) for waters with <5 μmol O_2_ kg^−1^, and CO_2-aq_ values up to 10–15 μmol kg^−1^ at the NO_2_^−^ maximum (5–7.5 μmol kg^−1^; Supplementary Fig. 2a).

During both years, suspended POC concentrations decreased by 15–20 μmol kg^−1^ over the upper 100 m of the water column and remained low (ca. 5 μmol kg^−1^) through the OMZ (Fig. 3i, j). Contrasting concentration profiles between both years were also observed. In 2015, surface POC was <18 μmol kg^−1^, whereas in 2018 it reached values up to 32 μmol kg^−1^ (Fig. 3i, j). These trends were also reflected in chlorophyll-a concentrations, which exhibited lower (< 5 μg L^−1^) values in the coastal area (Stn T1 and T2) in 2015, whereas higher (up to 11.8 μg L^−1^) and more spatially distributed concentrations (from Stn T1 to T3) were observed in surface waters during 2018 (Supplementary Fig. 3).

### Isotopic signature of DIC and POC

The stable isotope composition of DIC and POC from the cross-shelf transect near Iquique (20 °S; Stations T1–6) showed contrasting conditions between both sampling periods (Fig. 3). In 2015, isotopically depleted δ^13^C_DIC_ values (0.0 to −0.5 ‰) and high DIC concentrations (>2250 μmol kg^−1^) were observed within the AMZ, but particularly in the upper core region (e.g., Stn T3, 100–200 m; Fig. 3g). Isotopically depleted δ^13^C_DIC_ values were also observed at depth in 2018, but mostly associated with the coastal area (Stns T1 and T3), and between 100 and 400 m depth (Fig. 3h) along with the highest concentration of NO_2_^−^ (Fig. 3d). During 2018, we observed even more isotopically depleted δ^13^C_DIC_ values (−0.6 to −0.8 ‰) and slightly lower, yet still high, DIC concentrations (<2200 μmol kg^−1^) compared to 2015 (Fig. 3g, h). The observed relationship between DIC concentration and δ^13^C_DIC_ (Supplementary Fig. 2b) suggests that δ^13^C_DIC_ is primarily governed by biological uptake in oxygenated waters (>20 μmol kg^−1^) and by microbial respiration in low O_2_ waters (<20 μmol kg^−1^), with the most depleted δ^13^C values associated with low oxygen and high *p*CO_2_ waters.

The difference in δ^13^C_POC_ values between both years was larger than in δ^13^C_DIC_. The δδ^13^C_POC_ in the upper 50 m ranged between −23‰ and −27‰ in 2015 and from −18.5‰ to −20.5 ‰ in 2018, with the most positive δ^13^C_POC_ values in the euphotic zone (Fig. 3k, l). These values are within the reported range of ^13^C fractionation due to photosynthetic carbon fixation^25,26^. However, within the AMZ core, and lower deep oxycline, we observed contrasting differences in δδ^13^C_POC_ values between both years. During 2015, δ^13^C_POC_ values were as low as −32‰ (Fig. 3k), particularly at 300 m depth at Stn 2, and down to 400 m at Stn 6. Notably, even more isotopically depleted δ^13^C_POC_ values were detected in oxic deep waters below the AMZ and down to 1000 m (Stn 6; Fig. 3k). On the other side, during 2018, δ^13^C_POC_ values within the AMZ core and deeper waters were more isotopically enriched and ranged from −23 to −28 ‰ (Fig. 3l).

The estimated values of δ^13^C_CO2_, as well as the respective isotopic fractionation (**ε**) against ^13^C between CO_2_ and POC, and DIC and POC, show contrasting conditions both through the water column of each year and between both years, especially below the lower oxycline (down to 400 m depth; Supplementary Fig. 4). In 2015, from the lower deep oxycline down to 1000 m depth, **ε** values varied ~21–23‰ and ~32–33‰ for CO_2_-POC and DIC-POC, respectively (Supplementary Fig. 4c, e). In 2018, **ε** values were ~17‰ and <24‰ for CO_2_-POC and DIC-POC over almost the entire water column, respectively (Supplementary Fig. 4d, f).

### Calcium carbonate saturation state

**Fig. 5:**
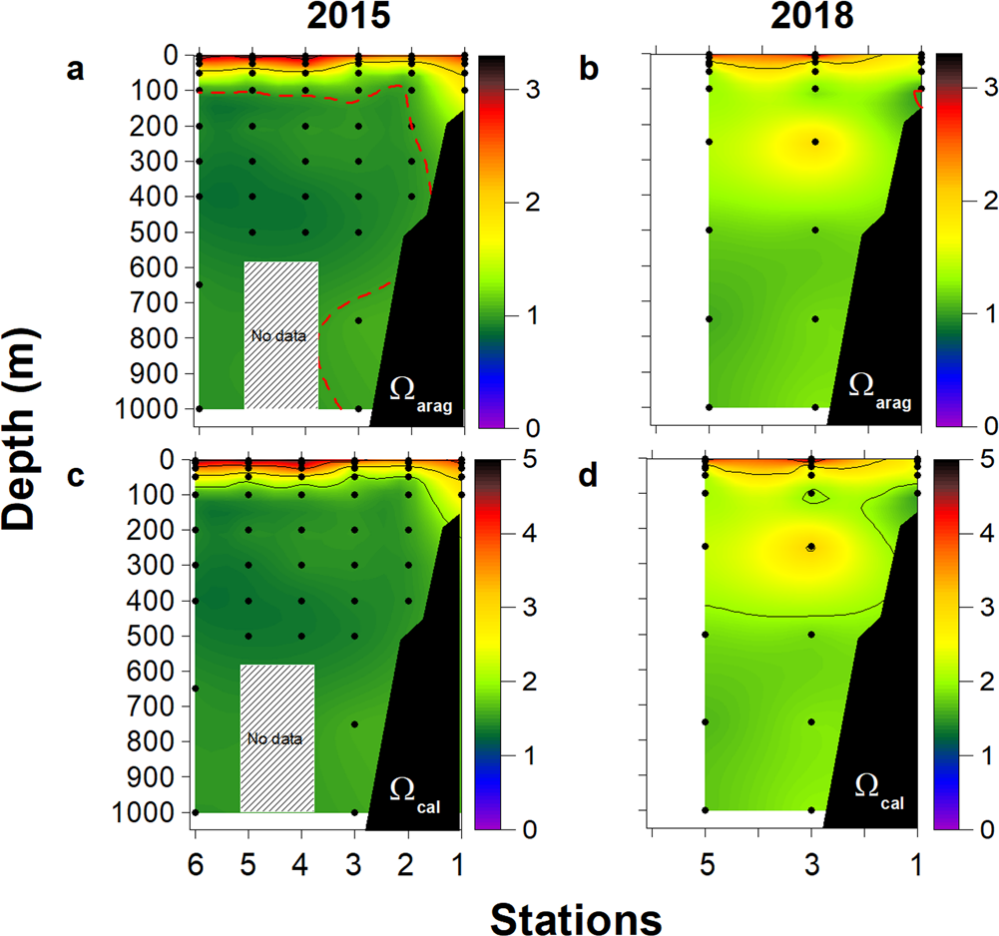
Calcium carbonate saturation state during both research cruises. Vertical cross-shelf sections of (**a**, **b**) aragonite saturation state (Ω_arag_) and (**c**, **d**) calcite saturation state (Ω_cal_) during research cruises in 2015 and 2018. The dotted red line for Ω values represents the isopleths of 1; below such value is considered CaCO_3_ undersaturation. The black dots represent sample locations. Gray box represents an area with non-collected data.

We observed distinct conditions for calcium carbonate saturation state (Ω) between both expeditions. During 2015, waters below 100 m were corrosive for aragonite, as depicted by Ω_arag_ < 1.0 (Fig. 5a) and low pH values < 7.8 (Fig. 2d). However, the calcite saturation horizon (Ω_calc_ < 1.0) was not observed in the upper 1000 m of the water column during both years (Fig. 5c, d). In 2015, both Ω_arag_ and Ω_calc_ were low at the lower deep oxycline between 350 and 450 m depth (Fig. 5a, c). Contrarily, both aragonite and calcite-undersaturated conditions were not observed in the water column in 2018, with a shoaling towards the coast (at 100 m depth in Stn 1), while Ω_arag_ values < 1.0 were observed in bottom waters (Fig. 5b).

### Model

**Fig. 6:**
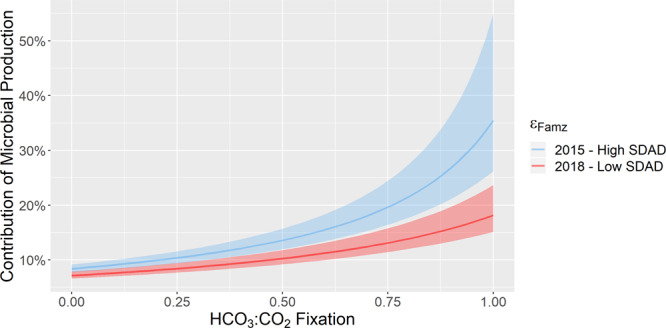
Summary results of carbon isotope model under range of inorganic carbon conditions and Low/High SDAD Scenarios. ε_Famz_ indicates the net fractionation factor based on a weighted average of each autotrophic metabolism expected to be active in the AMZ. Shaded areas denote error estimates in ε_Famz_ derived from the mean fractionation factors of individual autotrophic metabolisms.

We used a simple carbon isotope mass balance model to determine the potential sources of microbially produced organic matter within AMZ waters, and to estimate how much of this carbon is exported to deeper waters in this region. The results of our carbon isotope model demonstrate that the potential contribution of microbial production to carbon export varies greatly (~7–35%), which relies on a net fractionation factor (ε_Famz_) of carbon fixation via autotrophic production in the AMZ, and the relative proportion of carbon fixed as HCO_3_ vs CO_2_ (Fig. 6). By incorporating depth integrated carbon fixation rates of each prominent autotrophic metabolism in the AMZ (see Table 1), we applied a weighted average to estimate the net fractionation associated with AMZ autotrophy (ε_Famz_: see Table 2) under two contrasting scenarios. Since AMZ systems are temporally variable, the two scenarios reflect the different possible ranges of metabolic rates between distinct seasonal conditions in 2015 and 2018 (i.e. high SDAD vs low SDAD, respectively). The model results demonstrate a significant difference in the microbial contribution to organic carbon export below the AMZ between years, ranging from ~2-17% greater in 2015 than 2018 (Fig. 6). This marked difference in the potential microbial contribution to POC below the AMZ (as much as double in 2015 than 2018) appears to be largely driven by the systematically heavier δ^13^C_POC_ (~5‰ across all depths) in 2018, likely a result of an increased phytoplanktonic contribution to the organic carbon pool.

**Table 1:** Depth integrated carbon fixation rates (µmol C m^−2^ d^−1^) present in the AMZ of northern Chile incorporated in our isotope mass balance model for weighting of the ε_Famz_ variable.

Carbon fixation	Low light cyanobacteria	SDAD Bacteria	Nitrifying archaea/bacteria	Anammox bacteria
Min C-Fixation Rate	0.02	0.32^a^	1.5	0.085
Max C-Fixation Rate	0.87	8.4^b^	3.58	1.204
Mean C-Fixation Rate	0.31	4.36	2.54	0.644
Fractionation Factor (εF)	−17.3 ±1.2 ‰	−24.85 ± 0.45 ‰	−19.3 ± 0.3 ‰	−18.2 ± 2.58 ‰

Isotope mass balance model includes average rates for each metabolism, except sulfur-driven autotrophic denitrification (SDAD)

^a^Minimum carbon fixation rate applied for SDAD bacteria under Low SDAD scenario

^b^Maximum carbon fixation rate applied for SDAD bacteria under High SDAD scenario

**Table 2:** Variables incorporated into the calculation of *f*_onet_ across all inorganic carbon conditions and under two scenarios of relative carbon fixation rates.

Model variable	High SDAD scenario	Low SDAD scenario
^13^δC_OCamz_	−23‰	−17.5‰
^13^δC_OCamz_	−28.5‰	−23.5‰
ε_Fe_	−14.6 ‰	−8.6‰
ε_Rez_	1‰	1‰
$\epsilon_{\rm{Famz}}$^a^	−23.1 ± 0.55‰	−19.4 ± 0.77‰
ε_Ramz_	1‰	1‰
^13^δC_DICez_	−7.5‰	−8‰
^13^δC_DICamz_	−5.5 to −10.5‰	−5.8 to −11‰
R_ez_	0.95	0.95
R_amz_	0.90	0.90
Year	2015	2018

HCO_3_:CO_2_ utilization is the ratio of HCO_3_ utilization to CO_2_ utilization in carbon fixation

*ez* euphotic zone, *AMZ* anoxic marine zone, *OC* organic carbon, *DIC* dissolved inorganic carbon, ε fractionation factor (F-carbon fixation, R-respiration), *SDAD* sulfur-driven autotrophic denitrification, ^13^δC isotopic composition of carbon pool

^a^ε_Famz_ is based on a weighted average of fractionation factors dependent on the relative carbon fixation rates of each autotrophic metabolism present in the AMZ

The remineralization rates in the euphotic zone and AMZ have a significant impact on the calculations of microbial contribution; as values approach complete recycling of AMZ-produced carbon (AMZ Remineralization = 100%), the contribution of microbial production expectedly approaches 0% (see Eq. 3). Remineralization in the euphotic zone is kept at 95% in all conditions to reflect realistic degradation rates of surface-derived organic matter in this region at 300 m^27^, meaning that ~5% of what is produced in the surface is exported to the AMZ (see Table 2).

The fixation of HCO_3_^−^ vs CO_2_ in the euphotic zone has no effect between model runs; the ^13^δC_POC_ in the surface remains the same as the ε_Fez_ is adjusted to remain consistent with the measured isotopic composition of the organic carbon pools. However, the model also demonstrates that the form of inorganic carbon utilized in the AMZ has a significant impact on the isotopic composition of POC in the AMZ, and thus the estimated total microbial contribution (Fig. 6). By incorporating relative contributions determined from the total range of metabolic rates reported in the literature for each autotrophic process present in the ETSP AMZ, we estimate the microbial contribution to organic carbon export to range between ~7 and 18% in the 2018 cruise, and between ~9 to 35% in the 2015 cruise (Table 2 and Fig. 6).

## Discussion

Geochemical and stable isotope data from two expeditions to the AMZ off northern Chile with contrasting oceanographic regimes highlight the biogeochemical variability of this environment. The interaction of local processes, such as the strength and spatial variation of upwelling conditions^28^, and its interconnection with regional processes, such as the intensity and displacement of mesoscale eddies^29^, in addition to the advection of tropical waters, determine surface productivity and the variability of the zonal and vertical boundaries of the AMZ core. The latter can in turn influence carbon cycling and export in this region – i.e., the source and fate of different carbon pools through the water column. Previous studies in the area have shown the accumulation of nitrite (>0.5 μmol kg^−1^)^13^ in oxygen-free waters^30^ between 200 and 300 m depth along the coast (ca. 20–26 °S). Anoxia is maintained by both the remineralization of organic matter produced in surface waters and the low ventilation due to strong stratification^31,32^.

Anoxic waters were characterized by low pH and high *p*CO_2_ conditions (pH < 7.5 and *p*CO_2_ > 1200 µatm), which places the AMZ of the ETSP as one of the most oxygen-deficient and acidic systems worldwide^32–35^. Carbonate chemistry in the AMZ is also highly temporal and spatially variable, as reflected by the contrasting levels of DIC concentration and pH between austral spring 2015 and summer 2018. Our results evidence that the ETSP AMZ off the coast of northern Chile embodies one of the shallowest (~100–400 m depth), low pH, high *p*CO_2_ AMZ systems in the world^5^, similar to what has been observed off the Peruvian coast^29^. Indeed, the aragonite saturation depth can be as shallow as 100 m depth, as observed during austral spring in 2015. The position of the AMZ core is also dynamic, with an AMZ core farther offshore during 2015 and more restricted to the continental shelf in 2018.

Notably, during 2015 we observed a decrease in DIC and aqueous CO_2_ (CO_2-aq_) concentrations down to ~30 µmol kg^−1^ in the AMZ core relative to adjacent, similarly oxygen-depleted waters (Supplementary Fig. 2A). In the same study area, Paulmier et al.^12^ observed a similar carbon deficit associated with the OMZ, which they related to oxygen loss due to thermal mechanisms during OMZ water formation in the equatorial region, since a decrease in DIC is expected in warm regions due to a lowering of gas solubility. Indeed, when we analyze *p*CO_2_ data collected in the ETSP between 7 and 15 °S^29^ shows a steady reduction in *p*CO_2_ as the Equatorial Subsurface Waters (ESSW) move southward along the Peruvian coast. Here, we explore the hypothesis that microbial processes involved in sulfur and nitrogen cycling can exert an additional partial control for such DIC deficit. For instance, the generally increased DIC concentrations in high-N* and low-O_2_ waters suggests active organic matter respiration via nitrate reducers, with the canonical denitrification pathway responsible for part of the N* registered in both years (>–20 µmol kg^−1^), which was remarkably high during 2018 (up to –40 µmol kg^−1^). Thus, while organic matter remineralization is expected to reduce alkalinity due to proton production, the observed high A_T_ in both years could be explained by a net nitrate input from an external source feeding nitrate reducers and denitrifiers^36^, as expected to occur particularly during the upwelling-active period registered in 2018. Inversely, the low DIC concentrations in the O_2_-depleted and N-deficient waters observed in some stations (Stns 5-6) during 2015 could be associated with dark carbon fixation.

The δ^13^C_DIC_ and δ^13^C_POC_ data suggest that chemosynthetic processes, which could reduce *p*CO_2_ in the AMZ^36,37^, were likely more dominant during 2015. It is well known that AMZs host an active and diverse autotrophic and heterotrophic microbial community^10,13^. Indeed, the unusually depleted δ^13^C_POC_ values, along with the **ε** values for CO_2_/DIC and POC observed in the AMZ core in 2015, reinforce the idea that inorganic carbon fixation processes such as SDAD and anammox^20^, previously reported to be active in oxygen-deficient regions^38,39^, might significantly influence the carbonate chemistry of AMZs. However, the low carbon fixation rates estimated through anammox^15^ (∼0.0004 µmol C L^−1^ d^−1^; based on a C:N = 0.066/1) compared to SDAD (from 0.3 up to 20 µmol C L^−1^ d^−1^ in the ETSP off Peru and Chile; Supplementary Table 1), support the idea that SDAD, with a higher carbon isotopic fractionation factor (mean of −24.8‰^40^) than anammox (mean of −18.2‰^20^), could be the dominant chemolithoautotrophic process driving carbon fixation in the AMZ. Although some studies suggest that SDAD can only be important in coastal AMZ waters^41^, sulfide oxidation rates calculated for the same study area (i.e. although with sulfide concentrations higher than in situ conditions) were high (i.e. relative to anammox and denitrification rates), and strongly coupled to nitrate reduction^13^. High sulfide oxidation activity has also been linked to metagenome analysis in our study area, which indicates the occurrence of enriched sequences matching the genomes of sulfur-oxidizing endosymbionts lineages (i.e. like Candidatus *Ruthia magnifica*, Candidatus *Vesicomyosocius okutanii*, and SUP05 pelagic linage), especially during spring/summer conditions^10,13,16^, such as in our 2015 expedition (i.e. austral spring). It has been suggested that sulfide oxidation is mainly sustained by sulfate reduction; however, the undetectable sulfide accumulation in OMZ/AMZ waters seems to be associated with its immediate oxidation back to sulfate, or to different sulfur intermediate compounds^42^, as part of what has been described as a cryptic sulfur cycle^13^. Furthermore, offshore SDAD activity could also be supported by eddy-driven zonal advection (i.e. a mesoscale process particularly active for northern Chile^43^) of SUP05 species and hydrogen sulfide that is frequently accumulated in OMZ shelf waters^39,44^. Actually, mesoscale eddies in this region can persist for weeks or months^45^. Moreover, Stn T5 is only ~75–80 km offshore. Therefore, a conservative estimate of sulfide oxidation using the ranges reported by both Canfield et al.^13^ and Callbeck et al.^39^ off northern Chile and Peru, respectively, as well as mean anammox rates for the same area^46–50^, suggests that dark carbon fixation could remove from 0.12 up to 0.63 µmol C L^−1^ day^−1^ during the southward travel of the ESSW. A study in the ETNP OMZ off Mexico^51^ also reported high DIC assimilation at the OMZ core (6.4 µmol C L^−1^ d^−1^) in association with depleted δ^13^C_POC_ values (−30.3‰), which they assigned to anammox activity based on molecular biomarkers analyses. However, Cavan et al.^52^ already suggested that anammox is unlikely to be solely responsible for dark carbon fixation in OMZ waters offshore. As stated above, the low anammox rates reported for our study area suggest that this metabolism plays a rather neglectable role in the DIC deficit in the core AMZ, despite the fact that enough NH_4_^+^, the apparent limiting substrate, is produced in the ETSP off northern Chile (Supplementary Table 2). Our results provide a robust biologically mediated mechanism for the observed DIC variability found in AMZs (deficit or accumulation), although we cannot rule out additional processes (biological/physical).

Aerobic NH_4_^+^ oxidizers, considered to be the main chemoautotrophs in the ocean^53^, have an **ε** similar to anammox (mean of 19.3‰^54^), and they can support up to 50% of the total dark carbon fixation in the upper oxycline of the ETSP AMZ^55^. However, the contribution of this process to carbon cycling within the AMZ is likely neglectable, as its activity^55–57^ and the abundance and expression of genes that regulate this pathway^10,16,56^ decrease with depth in the AMZ. For instance, NH_4_^+^ oxidation rates drop three orders of magnitude in experiments using water from the oceanic AMZ oxycline (250 μmol O_2_ L^−1^; 800 nmol N L^−1^ d^−1^)^55^, the upper AMZ (<5 μmol O_2_ L^−1^; 150 nmol N L^−1^ d^−1^), and the AMZ core (6 nmol O_2_ L^−1^; 4 nmol N L^−1^ d^−1^)^57^. At depressed rates, NH_4_^+^ oxidizers fix 0.5 nmol C L^−1^ d^−1^ (based on a C:N ratio = 1.0:8.3)^58^, which is three orders of magnitude lower than the mean C fixation estimated for SDAD in the AMZ (see Supplementary Table 2). Nevertheless, since part of the particulate material settled to the AMZ could have the δ^13^C imprint from NH_4_^+^ oxidizers at the oxycline and/or at the upper OMZ boundary, its potential contribution to the organic carbon pool in the AMZ should be considered.

Increased A_T_ (>2300 µmol kg^−1^) and high DIC concentrations (>2200 µmol kg^−1^ in some samples) in O_2_-depleted and N-deficient waters suggest a predominance of heterotrophic denitrification over autotrophic processes during 2018. Furthermore, the **ε** values between CO_2_ and POC, as well as between DIC and POC, were significantly lower than in 2015 over almost the entire water column (17‰ and 24‰, respectively). Therefore, and considering the higher phytoplankton and POC biomass in surface waters during 2018, our results suggest that most (or at least a significant fraction) of the organic carbon exported to the AMZ and below derives from recently settled photosynthetically-fixed carbon in surface waters^25,26,59^. This fresh organic material might have stimulated the heterotrophic community, particularly the fast-growing denitrifiers^49,60^ (i.e. relative to the slow growing anammox bacteria^61^). This argument is also confirmed from previous studies in the same region^49,60^, where the highest denitrification rates were found associated with areas with high chlorophyll levels in the euphotic zone. In general, N cycling in the AMZ is tightly coupled to the organic matter produced and exported from the surface^60^.

The dominant microbial functional types found in the AMZ core of this region have the potential for sulfur/ammonium oxidation, nitrate/nitrite reduction, and especially inorganic carbon fixation (C1 carbon fixation)^10^, with denitrification, anammox, and sulfur oxidation pathways as the dominant processes in the core of the AMZ^10,13^. SUP05 and SAR324 are well-known bacterial clades with the metabolic flexibility to perform bicarbonate (DIC) uptake^13,62^ as well as methane oxidation^63^, and they are common in this oxygen-limited system^10^. ^15^N-tracer approaches have confirmed active N-loss through denitrification and anammox in this region^48,50,64^, although both processes exhibit high spatiotemporal variability in this and other AMZ systems^65–67^. In general, anammox, which is particularly predominant in the ETSP, is highly active just below the upper AMZ boundary, and declines exponentially with depth^48,49^. However, no depth dependence has been discerned for denitrification^49^. On the other hand, beyond oxygen limitation^47,68,69^, the input of electron donors exerts a main control of N-removal processes, i.e., organic matter quality and lability^60,70^, NH_4_^+^ availability from organic matter remineralization^46,48,56,60^, and sulfide readiness^13,38^. Thus, the observed variability in the dominance of different microbial processes between 2015 and 2018 is likely associated with the seasonal oceanographic dynamic reported for AMZ waters in this region^28,71^. Our 2015 sampling occurred during austral spring conditions and under a moderated upwelling regime with a narrow upwelling signal at the coast, whilst the 2018 sampling occurred during an intense summer upwelling event with high productivity in surface waters (20 mg Chl-a m^−3^).

The export of organic matter from dark carbon fixation processes occurring in AMZ waters to deeper layers of the ocean may contribute to the depleted δ^13^C_POC_ signatures found in waters below the AMZ in 2015. A recent organic geochemical study in the AMZ off northern Chile^72^ reported that a biomarker specific of anammox bacteria^73^ was not only abundant in the suspended organic matter within AMZ waters, but also in oxygenated waters below, possibly indicating the occurrence of exported biomass from the AMZ, and/or active anammox bacteria in anoxic microniches within sinking particles. However, the lack of robust biomarkers for SDAD makes a quantitative comparison of the relative contribution of anammox vs. SDAD to POC using biomarkers in AMZ regions still difficult.

Our carbon isotope-mixing model investigated the potential contribution of microbial autotrophs in AMZ waters based on the net isotopic fractionation factor (ε_Famz_) during carbon fixation. The model includes contributions from low light-adapted cyanobacteria, anammox bacteria, nitrifying archaea/bacteria, and sulfide-oxidizing bacteria during the two cruises. The carbon chemistry and C:N stoichiometries from the 2015 and 2018 cruises suggest a dynamic shift, in terms of carbon fixation, from a SDAD dominated AMZ to a largely photoautotrophic/nitrifying system. Thus, we applied the carbon isotope model under two distinct scenarios, a relatively high SDAD scenario to reflect the conditions in the 2015 cruise, and a low SDAD scenario in 2018 (Table 2). In order to constrain the potential range of total microbial contribution to carbon export, we compared depth-integrated metabolic rates of chemoautotrophy in the AMZ under these two generalized scenarios (Table 1). The model results indicate that the total microbial contribution from AMZ waters to carbon export likely ranged between ~7 and 18% in the 2018 cruise, and between ~9 and 35% in the 2015 cruise (Fig. 6). Our estimates provide quantitative evidence for a significant and novel secondary source of organic carbon exported to the mesopelagic region from oxygen-deficient waters that have been generally overlooked. Recently, using intact polar lipids in size-fractionated POM, Cantarero et al.^74^ suggested that the standing stock of microbial biomass in AMZ waters of this system might be larger than that of the oxic photic zone, thus supporting the notion that dark carbon fixation might contribute to carbon export to the deeper ocean. Indeed, Lengger et al.^75^ demonstrated that organic carbon produced through chemoautotrophic carbon fixation could constitute a substantial proportion (17%) of the organic matter exported from the Arabian Sea OMZ to underlying surface sediments.

It has been suggested that similar to episodes of transient warming in earth history, modern and future ocean warming could impact the oceanic biological pump, largely via increasing surface ocean temperature^76^, a reduction in the remineralization depth^77,78^, and therefore reduced CO_2_ storage^76^. Our results highlight the potential implications of enhanced microbial processes in AMZ waters^77^ as a source of isotopically depleted organic carbon that has not been considered when estimating changes in carbonate chemistry and carbon cycling in projections of AMZ expansion. Moreover, dark carbon fixation by microorganisms involved in nitrogen, carbon, and sulfur cycling could also influence, through dark carbon fixation, the carbonate chemistry conditions (pH, A_T_, and *p*CO_2_) for temporary or permanent AMZ inhabitants^79^. Thus, the recent evidence for decreasing pH due to reduced ventilation in the deep ocean^80^ deserves revision in some oceanic regions, since such conclusions do not consider the role of microbially mediated biogeochemical processes associated with sulfur and nitrogen cycles, which can modify the carbonate chemistry in AMZ waters at depth. In consequence, there is a need for new biogeochemical models that take into account sulfur/nitrogen-driven microbial processes occurring in AMZs under future scenarios of ocean change.

## Methods

### Field sampling and hydrographic profiles

Samples were collected during two expeditions to the ETSP off northern Chile during late November 2015 (LowpHOX 1) and February 2018 (LowpHOX 2) onboard the R/V *Cabo de Hornos*. The sampling design during 2015 considered an inshore-offshore transect off Iquique (20 °S) with six sampling stations (T1–T6), and a longitudinal transect with six sampling stations (L1–L6) from 20° down to 29.5 °S (Fig. 1). During 2018 we studied three sampling stations along the inshore-offshore transect (T1, T3, and T5).

Hydrographic profiles were recorded during both cruises using the same SeaBird SBE-911+ CTD system equipped with two temperature and conductivity sensors, a SBE 43 oxygen sensor and fluorometer, and 24 10-L General Oceanic Niskin Bottles. Temperature and conductivity sensors were pre-cruise calibrated. The raw data were processed following the recommended procedures provided by the manufacturer and were averaged into one dbar bins. Additionally, a pump profiling system (PPS) was also used for water collection and high-resolution O_2_ measurement.

### Upwelling index and MODIS images

The Upwelling Index is based on Ekman Transport estimates provided by NOAA ERDDAP (http://coastwatch.pfeg.noaa.gov/erddap/griddap/erdlasFnTran6.html). The horizontal resolution of the transport estimates is 1 × 1° latitude × longitude on a 6-h interval. We chose the grid point at 20.5 °S and 70.5°W offshore Iquique. The Upwelling Indices were calculated with an algorithm published by NOAA ERDDAP on their website.

The data to compute Sea Surface Temperature and Chlorophyll-a maps were obtained from the MODIS-Aqua website https://oceandata.sci.gsfc.nasa.gov/MODIS-Aqua/Mapped/8-Day/4km/. MODIS (or Moderate Resolution Imaging Spectroradiometer) is a key instrument aboard the Terra (originally known as EOS AM-1) and Aqua (originally known as EOS PM-1) satellites. Terra’s orbit around the Earth is timed so that it passes from north to south across the equator in the morning, while Aqua passes south to north over the equator in the afternoon. Terra MODIS and Aqua MODIS screen the Earth’s surface every 1–2 days, acquiring data in 36 spectral bands, or groups of wavelengths (see MODIS Technical Specifications). The horizontal resolution of the database is 4 × 4 km and 8-day composites of cloud-free ocean are provided.

### Chlorophyll a, dissolved nutrients, and elemental stoichiometry

Chlorophyll *a* concentration and dissolved inorganic N (nitrite, NO_2_^−^ and nitrate, NO_3_^−^) and phosphate (PO_4_^3−^) were analyzed using standard protocols^81,82^. In order to calculate the deviation from the classical Redfield ratio (N:P = 16:1) (Redfield et al. 1963), we calculated the tracer N* ^83^ (Eq. 1):

1 $${\mathrm{N}} \ast = (\left[ {{\mathrm{NO}}_3^ - } \right]-16 \times \left[ {{\mathrm{PO}}_4^{3 - }} \right] + 2.9) \times 0.87$$

where [NO_3_^−^] and [PO_4_^3−^] are the concentrations of nitrate and phosphate in μmol kg^−1^, respectively. Positive and negative N* are an indicator of an excess or deficit of NO_3_^−^relative to PO_4_^3−^ ^82^, respectively, and values are typically arbitrary units rather than absolute concentrations.

### Carbon pools and stable isotope analyses

Samples for pH_T_ were collected in 50 mL syringes and immediately transferred to a 25 mL thermostatted cell at 25.0 ± 0.1 °C for standardization, with a pH meter Metrohm® using a glass combined double junction Ag/AgCl electrode (Metrohm model 6.0258.600), which was calibrated with 8.089 Tris buffer solution as a certified reference material (CRM, supplied by Andrew Dickson, Scripps Institution of Oceanography, San Diego, USA) at 25 °C. pH values are reported on the total scale (pH_T_). Samples for total alkalinity (A_T_) were poisoned with 50 µL of saturated HgCl_2_ solution and stored in 500 mL borosilicate BOD bottles with ground-glass stoppers lightly coated with Apiezon L^®^ grease and kept in darkness and room temperature. A_T_ was determined using the open-cell titration method^84^, by using an automated Alkalinity Tritrator Model AS-ALK2 Apollo SciTech. All samples were analyzed at 25 °C ( ± 0.1 °C) with temperature regulation using a water-bath. Analytical accuracy was controlled against a certified reference material (CRM, supplied by Andrew Dickson, Scripps Institution of Oceanography, San Diego, USA) and the A_T_ repeatability averaged 2-3 µmol kg^−1^. DIC samples were collected in 250 mL Wheaton® glass bottles and preserved with 50 μL saturated HgCl_2_ solution. Immediately after opening the sample bottle, a digital syringe withdrew a small volume (0.5 ml), acidified it with 10% phosphoric acid and subsequently measured the evolved CO_2_ with a LICOR 6262 non-dispersive infrared gas analyzer. Certified seawater reference materials from A. Dickson were used to ensure the quality of DIC determination by preparing a calibration curve covering the range of DIC from 200–2000 μeq L^−1^, with a resulting precision averaging ≈ 0.1% (range 0.05–0.5%). Temperature, salinity, A_T,_ and DIC data were used to calculate aqueous CO_2_ (CO_2_-aq), *p*CO_2_, and Calcite Saturation State (Ω_calcite_) Analyses were performed using CO_2_SYS software for MS Excel^85^ set with Mehrbach solubility constants^86^ refitted by Dickson & Miller^87^. The KHSO_4_ equilibrium constant determined by Dickson^88^ was used for all calculations.

Stable isotopic (δ^13^C) measurement of DIC was performed following a method modified version of the method by Torres et al (2015)^89^. Seawater DIC samples were collected in 250 mL Wheaton® glass bottles and preserved with 50 μL saturated HgCl_2_ solution. Inside a glove bag, 1 mL water sample was transferred into pre-cleaned (10% HCl-rinsed and 540 °C/4 hrs combusted) 12 mL Exetainer® vials and then capped before sealing the vial with an ultra-high purity helium headspace. DIC samples were acidified with 50 µL 85% H_3_PO_4_ via syringe injection through the septum, and allowed to equilibrate at room temperature for 12 hours. Evolved headspace *p*CO_2_ δ^13^C_DIC_ was then measured using a Gas Bench II and a Finnigan Delta Plus isotope ratio mass spectrometer at UC Irvine, with an analytical uncertainty of ± 0.05‰.

In order to estimate the isotopic signature of CO_2_ incorporated during uptake, we also estimated the δ^13^C of ambient CO_2_(aq) by using the Eq. 2 derived from Rau et al.^90^:

2 $$\delta ^{13}{\mathrm{C}}_{{\mathrm{CO}}2} = \delta ^{13}{\mathrm{C}}_{{\mathrm{DIC}}} + 23.644-\left( {9701.5/{{T}}_{\mathrm{K}}} \right)$$

where δ ^13^C_DIC_ is the δ ^13^C of ambient DIC and *T*_K_ is the corresponding temperature in Kelvin scale.

Seawater samples (500–1000 mL) were collected by vacuum filtration through pre-combusted (4 h at 450 °C) MFS GF/F glass fiber filters (0.7 µm nominal pore size, 25 mm diameter) for POC analyses. Prior to filtration, filters were rinsed with 10% HCl followed by deionized water to eliminate any trace of inorganic carbon. In 2015 cruise, POC concentration and δ ^13^C_POC_ were determined at the Stable Isotope Facility at the University of California Davis by using an Elementar Vario EL Cuve or Micro Cube elemental analyzer (Elementar Analysensysteme GmbH, Hanau, Germany) interfaced to a PDZ Europa 20–20 isotope ratio mass spectrometer (Sercon Ltd., Cheshire, UK). The long-term precision reported is ±0.2‰ for δ ^13^C_POC_ in this range with precision decreasing on the lower end. POC concentration and the δ^13^C_POC_ for the 2018 cruise filtrates were measured using a Thermo Scientific Elemental Analyzer—Isotope Ratio Mass Spectrometer at the CU Boulder Earth Systems Stable Isotope Lab. Purified acetanilide, ethylenediaminetetraacetic acid, and in-house (Pugel) standards were measured for external calibration and drift corrections with a total carbon and nitrogen analytical precision between 1.1–2.2% and 0.6–2.2%, respectively, and a δ^13^C_POC_ analytical precision between 0.09 and 0.17‰ across all analysis runs. During analysis, samples are interspersed with several replicates of at least two different laboratory standards.

### Model

We utilized a carbon isotope mass balance model to determine potential sources of organic matter and estimate the impact of microbial dark autotrophic production to carbon export from the AMZ (Eqs. 3, 4 and 5); where ez is the euphotic zone, amz the anoxic marine zone, *Ф* represent the mass fluxes (P-primary productivity, R-respiration), *r*_*e*_ is the remineralization in the euphotic zone, *r*_*o*_ the remineralization in OMZ), δ is the isotopic composition of carbon pool, ε is the fractionation factor (F-carbon fixation, R-respiration), OC is the organic carbon, and DIC the dissolved inorganic carbon concentration. The exported carbon pool in the mesopelagic region is described as a ratio of fluxes between net-AMZ and -total net primary production (i.e. euphotic zone + AMZ net primary production; Eq. 3). Remineralization rates are defined as the relative fluxes of respiration/production (Eq. 4). The contribution of productivity in the AMZ to carbon export (*f*_onet_) is defined in terms of: the net productivity fluxes, the isotopic fractionation factors (ε) of carbon fixation and respiration in the euphotic zone and AMZ, and the isotopic composition of the organic and inorganic carbon pools in both the euphotic zone and AMZ (Eq. 5).

The measured δ^13^C of bulk organic matter and total dissolved inorganic carbon from both the euphotic and AMZs along the 2015 + 2018 oceanographic expeditions are fed into Eq. 3 under a range of inorganic carbon conditions and 2 different scenarios of metabolic activity. We calculated *f*_onet_ assuming different proportions of HCO_3_ and CO_2_ utilized in carbon fixation from AMZ autotrophy expressed as a ratio (HCO_3_:CO_2_ fixation) from 0 to 1. In addition, we calculated *f*_onet_ under two scenarios (High SDAD and Low SDAD) by estimating an ε_Famz_ value derived from a weighted average from depth integrated rates of carbon fixation from each autotrophic metabolism active in the AMZ (see Table 1 for summary of model conditions and scenarios).

3 $$f_{{\rm{onet}}} = \frac{{{{\Phi }}_{{\rm{amzP}}}{{\Phi }}_{{\rm{amzR}}}}}{{{{\Phi }}_{{\rm{amzP}}} - {{\Phi }}_{{\rm{amzR}}} + {{\Phi }}_{{\rm{ezP}}} - {{\Phi }}_{{\rm{ezR}}}}}$$

4 $$r_e = \frac{{{\upvarepsilon }}_{{\rm{Rez}}}}{{{\upvarepsilon }}_{{\rm{Fez}}}};\,r_o = \frac{{{\upvarepsilon }}_{{\rm{Ramz}}}}{{{\upvarepsilon }}_{{\rm{Famz}}}}$$

5 $$f_{{\rm{onet}}} = \frac{{(r_o - 1)(\delta _{{\rm{OCez}}} - \delta _{{\rm{OCamz}}})}}{{(r_o - 1)\delta _{{\rm{DICez}}} + \delta _{{\rm{DICamz}}} - {\it{\epsilon }}_{{\rm{Fez}}} + r_e{\it{\epsilon }}_{{\rm{Rez}}} + {\it{\epsilon }}_{{\rm{Famz}}} - r_o(\delta _{{\rm{OCamz}}} - {\it{\epsilon }}_{{\rm{Fez}}} + r_e{\it{\epsilon }}_{{\rm{Rez}}} + {\it{\epsilon }}_{{\rm{Ramz}}})}}$$

The autotrophic metabolisms present with significant carbon fixation rates within the AMZ in these model runs are limited to low light-adapted cyanobacteria, nitrifying bacteria/archaea, anammox bacteria, and sulfur-oxidizing bacteria. Average fractionation factors for each autotroph’s respective carbon fixation pathway^20,40,54,91,92^ are incorporated into the model (Table 1) and expressed as a weighted average (ε_Famz_; Table 2) based on the relative rates of carbon fixation associated with each respective metabolism.

We included averaged depth-integrated carbon fixation rates of anammox^46–50^; aerobic NH_4_^+^ oxidation^55^ and low-light-adapted cyanobacteria^93^, and min/max rates of sulfide oxidation^39^ (i.e. corresponding to low/high SDAD scenarios, respectively). Depth integrated NH_4_^+^ oxidation rates were estimated from discrete carbon fixation rates off northern Chile by Molina & Farías^55^, assuming nitrifying bacteria/archaea to be limited to ~100 m of the lower oxycline and upper OMZ. Sulfate-reducing bacteria in the ETSP are thought to be predominantly heterotrophic and provide a significant source of mineralized NH_4_^+^ for anammox bacteria^13^, but are not expected to be a significant source of fixed carbon and are thereby not included in this calculation.

## Supplementary information

Supplementary Information

## Data Availability

The data that support the findings of this study are available from the corresponding author upon reasonable request

## References

[1] Gilly WF (2013). Oceanographic and biological effects of shoaling of the oxygen minimum zone. Annu. Rev. Mar. Sci..

[2] Long, M. C., Deutsch, C. & Ito, T. Finding forced trends in oceanic oxygen. *Global Biogeochem. Cycles* **30**, 10.1002/2015GB005310 (2016).

[3] Wyrtki K (1962). The oxygen minima in relation to ocean circulation. Deep-Sea Res..

[4] Stramma L (2010). Eastern Pacific oxygen minimum zones: Supply paths and multidecadal changes. J. Geophys. Res..

[5] Paulmier A, Ruiz-Pino D (2009). Oxygen minimum zones (OMZs) in the modern ocean. Progr. Oceanogr..

[6] Whitney FA, Freeland HJ, Robert M (2007). Persistently declining oxygen levels in the interior waters of the eastern subarctic Pacific. Progr. Oceanogr..

[7] Stramma L (2008). Expanding oxygen-minimum zones in the tropical oceans. Science.

[8] Emerson S (2004). Temporal trends in apparent oxygen utilization in the upper pycnocline of the North Pacific: 1980–2000. J. Oceanogr..

[9] Revsbech NP (2009). Determination of ultra-low oxygen concentrations in oxygen minimum zones by the STOX sensor. Limnol. Oceanogr. Methods.

[10] Ulloa O (2012). Microbial oceanography of anoxic oxygen minimum zones. Proc. Natl Acad. Sci. U. S. A..

[11] Padilla CC (2017). Metagenomic binning recovers a transcriptionally active gammaproteobacterium linking methanotrophy to partial denitrification in an anoxic oxygen minimum zone. Front. Mar. Sci..

[12] Paulmier A, Ruiz-Pino D, Garçon V (2011). CO_2_ maximum in the oxygen minimum zone (OMZ). Biogeosciences.

[13] Canfield DE (2010). A cryptic sulfur cycle in oxygen-minimum-zone waters off the Chilean coast. Science.

[14] Naqvi SWA (1998). Budgetary and biogeochemical implications of N_2_O isotope signatures in the Arabian Sea. Nature.

[15] Koeve W, Kähler P (2010). Heterotrophic denitrification vs. Autotrophic anammox—quantifying collateral effects on the oceanic carbon cycle. Biogeosciences.

[16] Stewart FJ, Ulloa O, DeLong EF (2012). Microbial metatranscriptomics in a permanent marine oxygen minimum zone. Environ. Microbiol..

[17] Ulloa, O. et al. in *The Prokaryotes—Prokaryotic Communities and Ecophysiology* (eds Rosenberg, E. et al.) (Springer-Verlag) 10.1007/978-3-642-30123-0_45 (2013).

[18] Keil RG (2016). A multiproxy approach to understanding the “enhanced” flux of organic matter through the oxygen-deficient waters of the Arabian Sea. Biogeosciences.

[19] Santos GM (2011). δ^14^C and δ^13^C of seawater DIC as tracers of coastal upwelling: a 5-year time series from Southern California. Radiocarbon.

[20] Schouten S (2004). Stable carbon isotopic fractionations associated with inorganic carbon fixation by anaerobic ammonium-oxidizing bacteria. Appl. Environ. Microbiol..

[21] Feely RA (2010). The combined effects of ocean acidification, mixing, and respiration on pH and carbonate saturation in an urbanized estuary. Est. Coast. Shelf. Sci..

[22] Brewer P, Peltzer ET (2009). Limits to Marine Life. Science.

[23] Franco AC (2018). Contrasting impact of future CO_2_ emission scenarios on the extent of CaCO_3_ mineral undersaturation in the Humboldt Current System. J. Geophys. Res..

[24] Schneider W (2003). Characteristics and formation of eastern South Pacific Intermediate Water. Geophy. Res. Lett..

[25] Fogel, M. L. & Cifuentes, L. A. in *Organic Geochemistry* (eds Engel, M. H. & Macko; S. A.) 73–98 (Plenum Press, 1993).

[26] Druffel ERM (2003). Penetration of anthropogenic carbon into organic particles of the deep ocean. Geophys. Res. Lett..

[27] Pantoja S, Sepúlveda J, González HE (2004). Decomposition of sinking proteinaceous material during fall in the oxygen minimum zone off northern Chile. Deep-Sea Res. I.

[28] Herrera L, Escribano R (2006). Factors structuring the phytoplankton community in the upwelling site off El Loa River in northern Chile. J. Mar. Syst..

[29] Hernández-Ayon, J. M. et al. Dynamics of the carbonate system across the peruvian oxygen minimum zone. *Front. Mar. Sci*. **6**, 617 10.3389/fmars.2019.00617 (2019)

[30] Thamdrup B, Dalsgaard T, Revsbech NP (2012). Widespread functional anoxia in the oxygen minimum zone of the eastern South Pacific. Deep Sea Res. I.

[31] Fiedler PC, Mendelssohn R, Palacios DM, Bograd SJ (2013). Pycnocline variations in the Eastern Tropical and North Pacific, 1958–2008. J. Clim..

[32] Franco AC (2014). Air-sea CO_2_ fluxes above the stratified oxygen minimum zone in the coastal region off Mexico. J. Geophys. Res..

[33] Feely RA (2008). Evidence for upwelling of corrosive “acidified” water onto the continental shelf. Science.

[34] Hoffman GE (2011). High-frequency dynamics of ocean pH: a multi-ecosystem comparison. PLOS ONE.

[35] Wang ZA (2013). The marine inorganic carbon system along the Gulf of Mexico and Atlantic coasts of the United States: Insights from a transregional coastal carbon study. Limnol. Oceanogr..

[36] Hu X, Cai W-J (2011). An assessment of ocean margin anaerobic processes on oceanic alkalinity budget. Glob. Biogeochem. Cycles.

[37] Reinthaler T, van Aken HM, Herndl GJ (2010). Major contribution of autotrophy to microbial carbon cycling in the deep North Atlantic’s interior. Deep-Sea Res. II.

[38] Galán A (2014). Temporal dynamics of nitrogen loss in the coastal upwelling ecosystem off central Chile: evidence of autotrophic denitrification through sulfide oxidation. Limnol. Oceanogr..

[39] Callbeck CM (2018). Oxygen minimum zone cryptic sulfur cycling sustained by offshore transport of key sulfur oxidizing bacteria. Nat. Commun..

[40] Ruby EG, Holger WJ, Deuser WG (1987). Fractionation of stable carbon isotopes during chemoautotrophic growth of sulfur-oxidizing bacteria. Appl. Environ. Microbiol..

[41] Löscher CR (2016). Water column biogeochemistry of oxygen minimum zones in the eastern tropical North Atlantic and eastern tropical South Pacific oceans. Biogeosciences.

[42] van Vliet, D. M. et al. The bacterial sulfur cycle in expanding dysoxic and euxinic marine waters. *Environ. Microbiol*. 10.1111/1462-2920.15265 (2020) 10.1111/1462-2920.15265 PMC8359478 33000514

[43] Wang Y (2018). Impact of mesoscale eddies on chlorophyll variability off the coast of Chile. PLoS ONE.

[44] Schunck H (2013). Giant hydrogen sulfide plume in the oxygen minimum zone off peru supports chemolithoautotrophy. PLoS ONE.

[45] Chaigneau A, Gizolme A, Grados C (2008). Mesoscale eddies off Peru in altimeter records: Identification algorithms and eddy spatio‐temporal patterns. Prog. Oceanogr..

[46] Hamersley MR (2007). Anaerobic ammonium oxidation in the Peruvian oxygen minimum zone. Limnol. Oceanogr..

[47] Kalvelage T (2011). Oxygen sensitivity of anammox and coupled N-cycle processes in oxygen minimum zones. PLoS ONE.

[48] Galán A (2009). Anammox bacteria and the anaerobic oxidation of ammonium in the oxygen minimum zone off northern Chile. Deep-Sea Res. II.

[49] Dalsgaard T (2012). Anammox and denitrification in the oxygen minimum zone of the eastern South Pacific. Limnol. Oceanogr..

[50] Dalsgaard T (2014). Oxygen at nanomolar levels reversibly suppresses process rates and gene expression in anammox and denitrification in the oxygen minimum zone off northern Chile. mBio.

[51] Podlaska A, Wakeham SG, Fanning KA, Taylor GT (2012). Microbial community structure and productivity in the oxygen minimum zone of the eastern tropical North Pacific. Deep Sea Res. I.

[52] Cavan E (2017). Remineralization of particulate organic carbon in an ocean oxygen minimum zone. Nat. Commun..

[53] Middelburg JJ (2011). Chemoautotrophy in the ocean. Geophys. Res. Lett..

[54] Könneke M, Lipp JS, Hinrichs K-U (2012). Carbon isotope fractionation by the marine ammonia-oxidizing archaeon *Nitrosopumilus maritimus*. Org. Geochem..

[55] Molina V, Farías L (2009). Aerobic ammonium oxidation in the oxycline and oxygen minimum zone of the eastern tropical South Pacific off northern Chile (<20 °S). Deep Sea Res. II.

[56] Lam P (2009). Revising the nitrogen cycle in the Peruvian oxygen minimum zone. Proc. Natl Acad. Sci. USA.

[57] Bristow LA (2016). Ammonium and nitrite oxidation at nanomolar oxygen concentrations in oxygen minimum zone waters. Proc. Natl Acad. Sci. USA.

[58] Joye SB (1999). Oxidation of ammonia and methane in an alkaline, saline lake. Limnol. Oceanogr..

[59] Hulthe G, Hulth S, Hall P (1998). Effect of oxygen on degradation rate of refractory and labile organic matter in continental margin sediments. Geochim. Cosmoch. Acta.

[60] Kalvelage T (2013). Nitrogen cycling driven by organic matter export in the South Pacific oxygen minimum zone. Nat. Geosci..

[61] Kuenen JG (2008). Anammox bacteria: from discovery to application. Nat. Rev. Microbiol..

[62] Swan BK (2011). Potential for chemolithoautotrophy among ubiquitous bacteria lineages in the dark ocean. Science.

[63] Sheik CS, Jain S, Dick GJ (2014). Metabolic flexibility of enigmatic SAR324 revealed through metagenomics and metatranscriptomics. Environ. Microbiol..

[64] Thamdrup B (2006). Anaerobic ammonium oxidation in the oxygen-deficient waters off northern Chile. Limnol. Oceanogr..

[65] Lam P, Kuypers MM (2011). Microbial nitrogen cycling processes in oxygen minimum zones. Annu. Rev. Mar. Sci..

[66] Zehr JP, Kudela RM (2011). Nitrogen cycle of the open ocean: from genes to ecosystems. Annu. Rev. Mar. Sci..

[67] Pajares MS, Ramos R (2019). Processes and Microorganisms Involved in the Marine Nitrogen Cycle: Knowledge and Gaps. Front. Mar. Sci..

[68] Smethie JR, William M (1987). Nutrient regeneration and denitrification in low oxygen fjords. Deep Sea Res. A: Oceanographic Res. Pap..

[69] Jensen MM, Kuypers MM, Lavik G, Thamdrup B (2008). Rates and regulation of anaerobic ammonium oxidation and denitrification in the Black Sea. Limnol. Oceanogr..

[70] Chang BX (2014). The effect of organic carbon on fixed nitrogen loss in the eastern tropical South Pacific and Arabian Sea oxygen deficient zones. Limnol. Oceanogr..

[71] Guiñez M, Valdéz J, Sifeddine A (2010). Spatial and temporal variability of the sedimentary organic matter associated with the Oxygen Minimum Zone (OMZ) in a coastal environment of the northern Humboldt Currrent, Mejillones Bay, Chile. Lat. Am. J. Aquat. Res..

[72] Matys ED (2017). Bacteriohopanepolyols along redox gradients in the Humboldt Current System off northern Chile. Geobiology.

[73] Rush D (2014). Anaerobic ammonium-oxidizing bacteria: a biological source of the bacteriohopanetetrol stereoisomer in marine sediments. Geochim. Cosmochim. Acta.

[74] Cantarero SI (2020). Size-fractioned contribution of microbial biomass to suspended organic matter in the Eastern Tropical South Pacific Oxygen Minimum Zone. Front. Mar. Sci..

[75] Lengger (2019). Dark carbon fixation in the Arabian Sea oxygen minimum zone contributes to sedimentary organic carbon (SOM). Glob. Biogeochem. Cycles.

[76] Marsay (2015). Attenuation of sinking particulate organic carbon flux through the mesopelagic ocean. Proc. Natl Acad. Sci. USA.

[77] Meyer KM, Ridgwell A, Payne JL (2016). The influence of the biological pump on ocean chemistry: implications for long-term trends in marine redox chemistry, the global carbon cycle, and the evolution of marine animal ecosystems. Geobiology.

[78] Capone DG, Hutchins DA (2013). Microbial biogeochemistry of coastal upwelling regimes in a changing ocean. Nat. Geosci..

[79] Riquelme-Bugueño R (2020). Diel vertical migration into anoxic and high-*p*CO_2_ waters: acoustic and net-based krill observations in the Humboldt Current. Sci. Rep..

[80] Chen A (2017). Deep oceans may acidify faster tan anticipated due to global warming. Nat. Clim. Change.

[81] Parsons, T. R., Maita, Y. & Lalli, C. M. A *Manual of Chemical and Biological Methods for Seawater Analysis* 173 (Pergamon Press,1984).

[82] Grasshoff, K., Kremling, K., & Ehrhardt, M. *Methods of Seawater Analysis* 3rd edn (Wiley– VCH, 1999).

[83] Gruber N, Sarmiento JL (1997). Global patterns of marine nitrogen fixation and denitrification. Glob. Biogeochem. Cycles.

[84] Dickson AG, Sabine CL, Christian JR (2007). Guide to best practices for ocean CO_2_ measurements. PICES Spec. Publ..

[85] Pierrot, D., Lewis, E. & Wallace, D. W. R. in *ORNL/CDIAC-105a* (Carbon Dioxide Information Analysis Center, Oak Ridge National Laboratory, U.S. Department of Energy, 2006).

[86] Mehrbach C (1973). Measurement of the apparent dissociation constants of carbonic acid in seawater at atmospheric pressure. Limnol. Oceanogr..

[87] Dickson AG, Millero FJ (1987). A comparison of the equilibrium constants for the dissociation of carbonic acid in seawater media. Deep Sea Res. I.

[88] Dickson AG (1990). Standard potential of the reaction: AgCl(s) + 1.2H_2_(g) = Ag(s) + HCl (aq), and the standard acidity constant of the ion HSO^−4^ in synthetic sea water from 273.15 to 318.15°K. J. Chem. Thermodyn..

[89] Torres ME, Mix AC, Rugh WD (2005). Precise δ^13^C analysis of dissolved inorganic carbon in natural waters using automated headspace sampling and continuous-flow mass spectrometry. Limnol. Oceanogr. Methods.

[90] Rau GH, Riebesell U, Wolf-Gladrow DA (1996). A model of photosynthetic ^13^C fractionation by marine phytoplankton based on diffusive molecular CO_2_ uptake. Mar. Ecol. Prog. Ser..

[91] Popp BN (1998). Effect of phytoplankton cell geometry on carbon isotopic fractionation. Geochim. Cosmochim. Acta.

[92] Sakata S (2008). Stable carbon-isotopic compositions of lipids isolated from the ammonia-oxidizing chemoautotroph *Nitrosomonas europaea*. Org. Geochem..

[93] Garcia-Robledo et al. Cryptic oxygen cycling in anoxic marine zones. *Proc. Natl. Acad. Sci. USA* **114**, 8319–8324 (2017). 10.1073/pnas.1619844114 PMC5547588 28716941

