# Supplementary material for: A source of isotopically light organic carbon in a low-pH anoxic marine zone
Source: Nat Commun. 2021 Mar 11;12:1604. doi: 10.1038/s41467-021-21871-4 (PMC7952585; doi:10.1038/s41467-021-21871-4)
Supplement: Supplementary file 1 — Supplementary Information [file 41467_2021_21871_MOESM1_ESM.pdf]

Supplementary Information

A source of isotopically light organic carbon in a low-pH anoxic marine zone

Cristian A. Vargas[1,2,3], Sebastian I. Cantarero[4], Julio Sepúlveda[2,4], Alexander Galán[5,6], Ricardo De Pol-Holz[7]; Brett Walker[8], Wolfgang Schneider[2,9], Laura Farías[3,9,10], Marcela Cornejo D'Ottone[2,11], Jennifer Walker[12], Xiaomei Xu[12] & Joe Salisbury[13]

Content:

- Supplementary Table 1 – 2
- Supplementary Figures 1 – 4
- Carbon Isotope Calculations

Supplementary Table 1. Dark carbon fixation rates by both Sulfur-driven Autotrophic Denitrification Rate and Anammox in the ETSP and ESP* OMZ region based on different studies in the region both offshore and inshore.

Sulfur-driven autotrophic denitrification rate (SDAD)				
Area	Specific Location	Range of carbon fixation rate (μmol C L^{-1} d^{-1})	Mean carbon fixation rate (μmol C L^{-1} d^{-1})	References
ETSP	Offshore Iquique, Chile (20ºS)	0.154 – 0.658	0.66**	Canfield et al. 2010
ETSP	Continental shelf off Peru	10 – 30	~20	Schunck et al. 2013
ETSP	Shelf and offshore Peru	0.077 – 0.592	0.33	Callbeck et al, 2018
*Mean SADR rate****			0.5 μmol C L^{-1} d^{-1}	(Based on Canfield et al. 2010 and Callbeck et al. 2018)
*Mean Anammox rate*****		0.00006 – 0.0006	0.0004 ***	
**Total potential Dark carbon fixation rate**		**0.12 – 0.63 μmol C L^{-1} d^{-1}**	**0.5 μmol C L^{-1} d^{-1}**	

*Eastern South Pacific

**By using a conversion ratio $H_2S:CO_2$ = 1:0.14 (Jorgensen et al. 1991)

*** We excluded the extreme carbon fixation rate reported by Schunck et al. 2013

****Based on a C:N= 0.07/1.3 (Kove & Kähler 2010)

Supplementary Table 2. Anammox demand and potential NH_4^+ sources in the ETSP OMZ region calculated with the unit of µmol N L^{-1} d^{-1} from different studies in the region.

Anammox Demand			
Area	Specific Location	Anammox Rate (nmol N L^{-1} d^{-1})	References
ETSP	Off Peru	1.1	Hamersley et al. 2007
ETSP	Off Peru	12.3	Kalevelage et al. 2011
ETSP	Off Iquique, Chile	3.9	Galan et al. 2009
ETSP	Off Iquique, Chile	3.6	Dalsgaard et al. 2012
ETSP	Off Iquique, Chile	12	Dalsgaard et al. 2014
Mean rate		**6.5 (= 0.0065 µmol N L^{-1} d^{-1})**	

NH_4^+ sources			
Area	Specific Location	Rates (µmol N L^{-1} d^{-1})	References
NO_3 reduction	Off Peru	0.01	Lam et al. 2009
DNRA	Off Peru	0.15	Lam et al. 2009
Other sources	Off Peru	0.05	Lam et al. 2009
Zooplankton excretion	Off Peru	0.08	Kiko et al. 2016 (excretion)* + Tutasi & Escribano 2020** (abundance)***
Mean rate		**0.30 µmol N L^{-1} d^{-1}**	

*Kiko, R., Hauss, H., Buchholz, F. & Melzner, F. Ammonium excretion and oxygen respiration of tropical copepods and euphausiids exposed to oxygen minimum zone conditions. *Biogeosciences* **13**, 2341 – 2255 (2016).

Tutasi, P. & Escribano, R. Zooplankton diel vertical migration and downward C flux into the oxygen minimum zone in the highly productive upwelling region off northern Chile. *Biogeosciences* **17, 455 – 473 (2020)

***Estimated by using depresses metabolic rates mimicking OMZ conditions (low pH/low O_2/low Tº) by Kiko et al. (2016) and zooplankton abundance during LowpHOX 1 cruise (2015) by Tutasi & Escribano (2020)

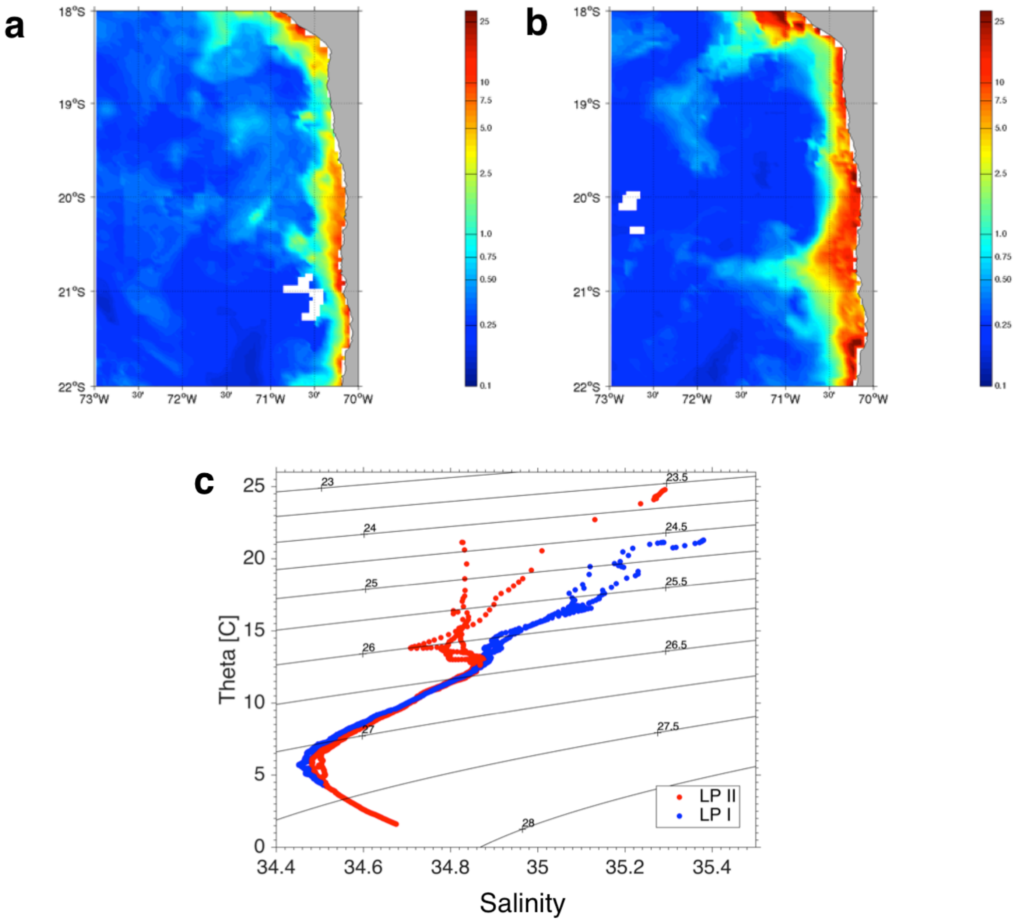

Supplementary Fig. 1: Physical oceanographic conditions during both expeditions.

(**a**) 25 November – 2 December 2015 composite of MODIS-Agua Chl-a for 2015 cruise; units are in mg m^{-3}; persisting clouds appear in white; (**b**) 2-9 February 2018 composite of MODIS-Agua Chl-a for 2018 cruise; units are in mg m^{-3}; persisting clouds appear in white; (**c**) Potential Temperature/Salinity diagram for the stations T1, T3 and T5. The blue dots correspond to 2015, the red dots to 2018 research cruise.

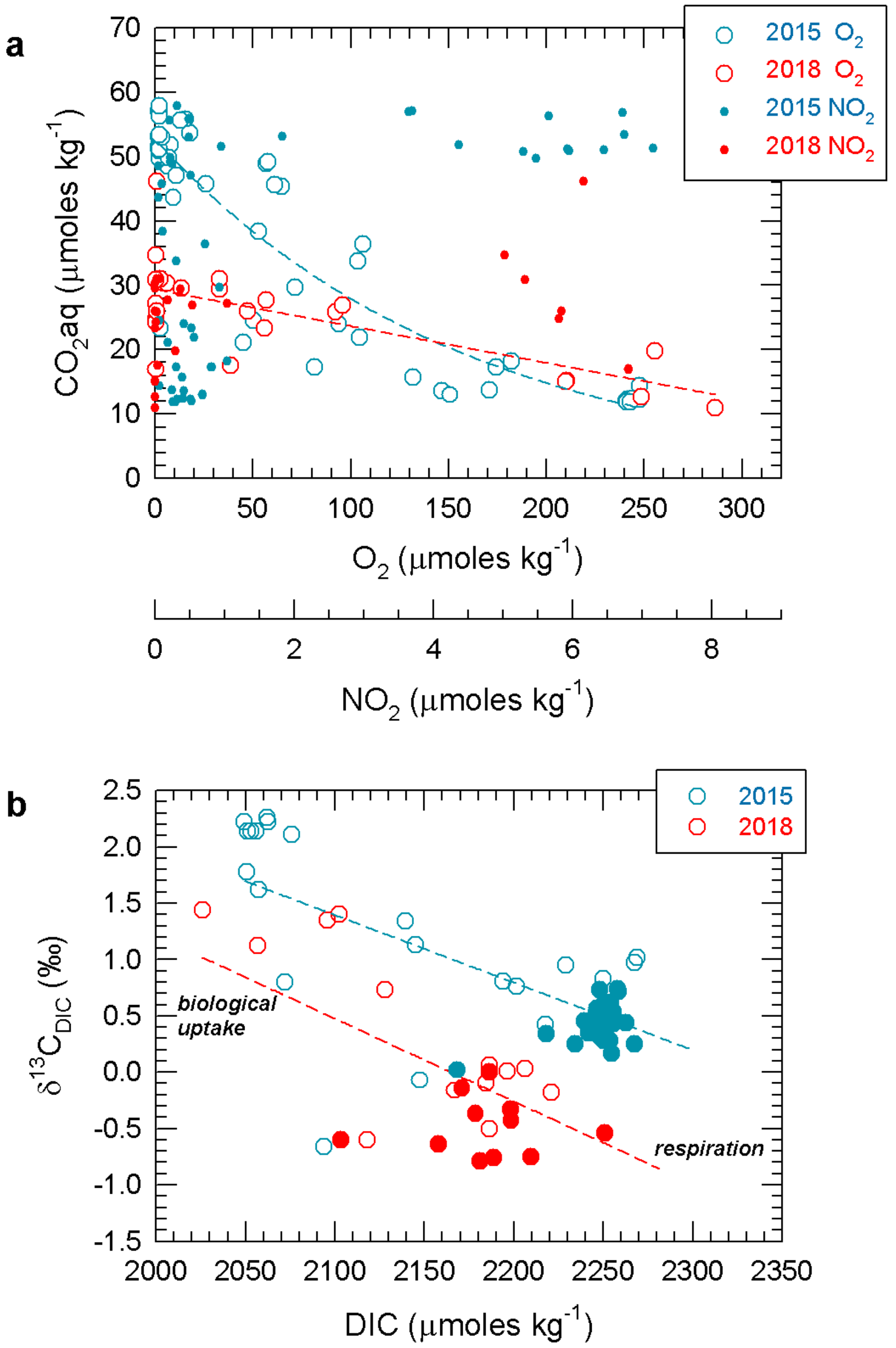

Supplementary Fig. 2: Analysis of aqueous CO_2 concentration and $\delta^{13}C_{DIC}$ in oxic and anoxic waters. (**a**) Relationship between dissolved oxygen and NO_2 concentration versus aqueous CO_2 and (**b**) Relationship between dissolved inorganic carbon (DIC) and $\delta^{13}C_{DIC}$ (‰) in oxic and in the core of the AMZ during research cruises. Results in (**a**) reveal the wide range of CO_2-aq levels for suboxic and/or anoxic waters (< 0.02 µmoles O_2, and > 4 µM NO_2) in the main AMZ core. Filled symbols in (**b**) correspond to data points associated with O_2 concentrations < 20 µmol kg^{-1}.

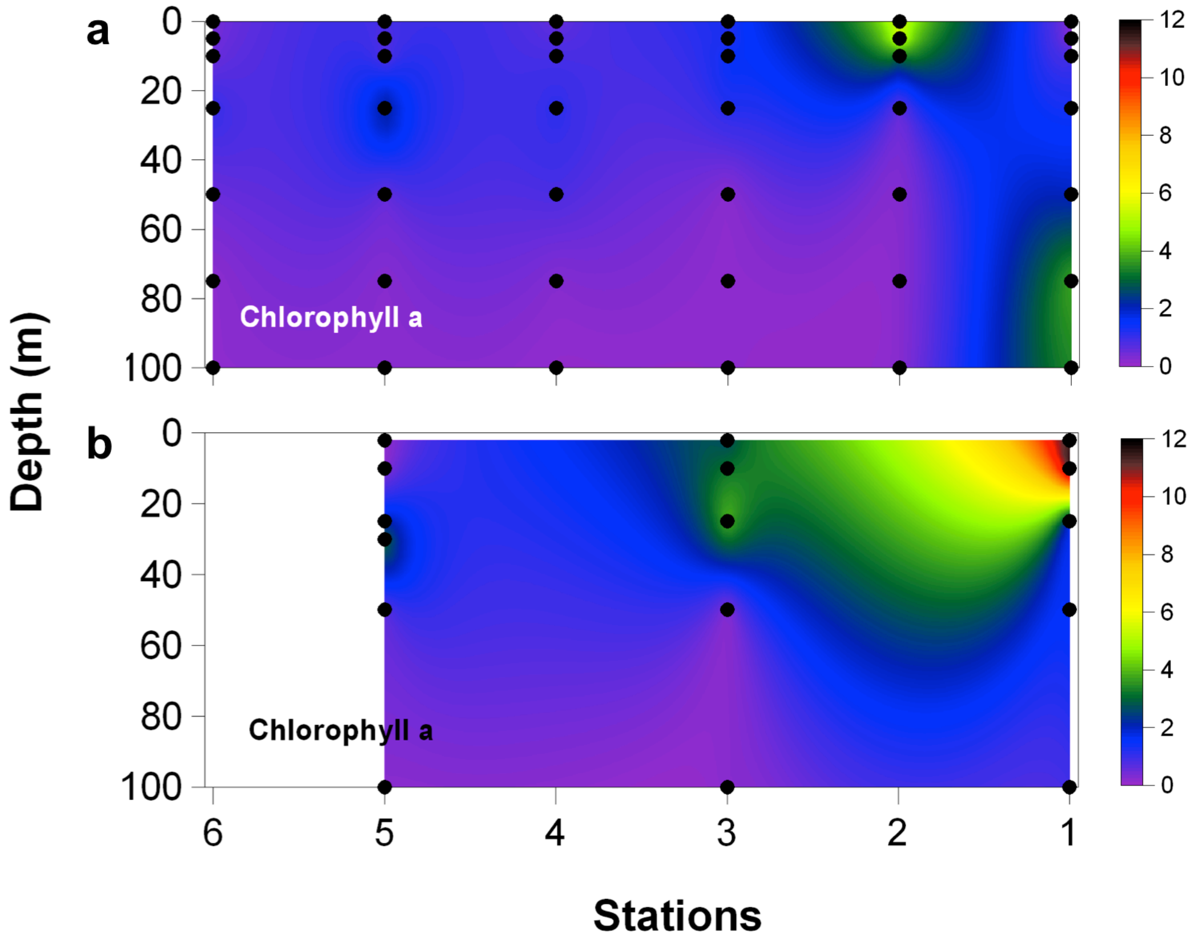

Supplementary Fig. 3: Vertical cross-shelf sections of chlorophyll *a* concentration in the upper 100 m depth during research cruises in (a) 2015 and (b) 2018. The black dots represent sample locations.

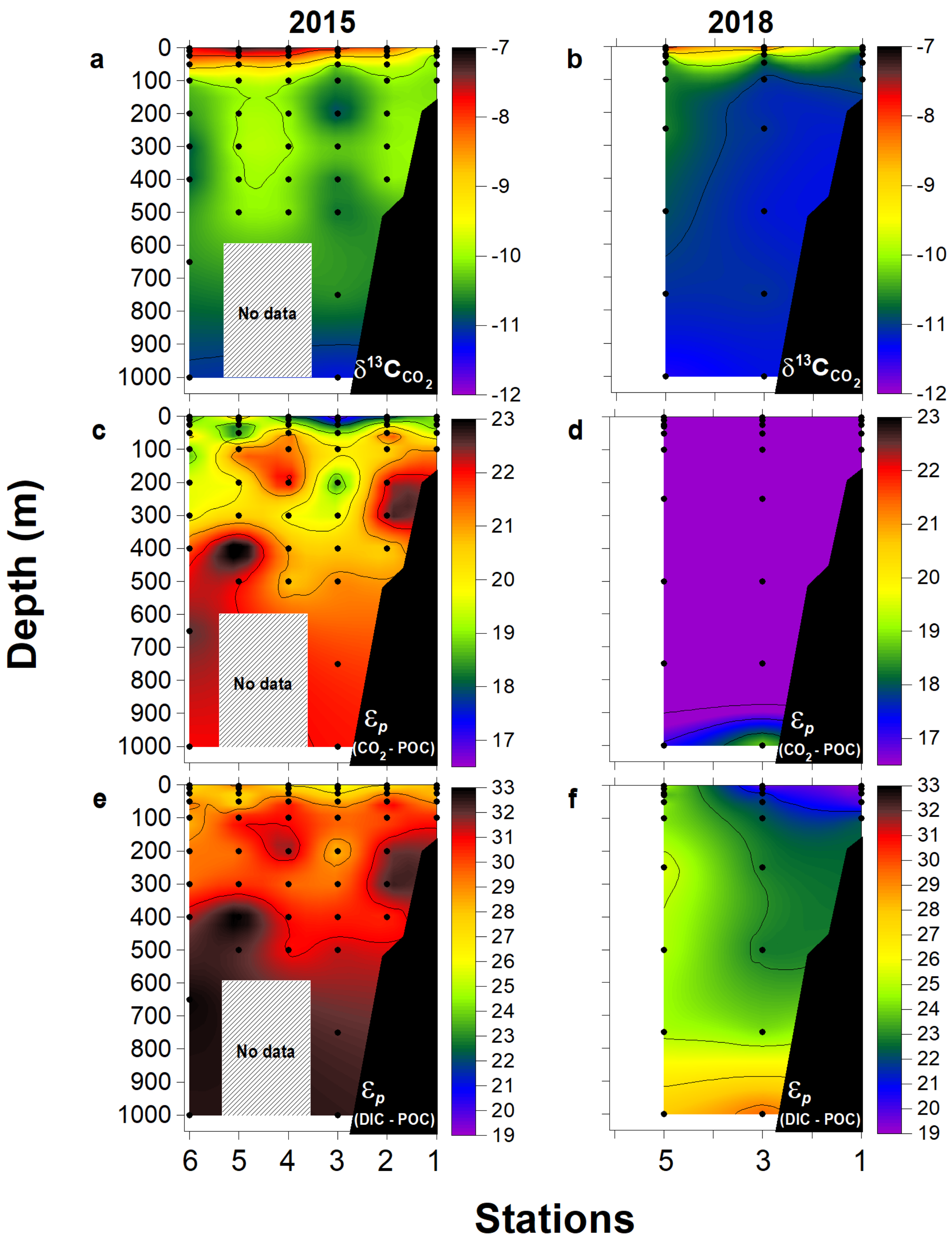

Supplementary Fig. 4: Carbon fractionation in the water column. Vertical cross-shelf sections of (**a**, **b**) isotopic signature of aqueous CO_2 and isotopic fractionation (ε) between (**c**, **d**) CO_2-aq and POC, and (**e**, **f**) DIC and POC during research cruises in 2015 and 2018. The black dots represent sample locations. Grey box represent area with non-collected data.

A key assumption in model Conditions 1 and 2 is that CO_2 is the only form of inorganic carbon fixed by autotrophs in the AMZ. While we expect this to be true for anammox bacteria (Kuenen, 2008), carbon fixation is likely more heterogeneous in cyanobacteria and sulfur cycling bacteria. Due to elevated pCO_2 (2-3 times higher in the AMZ than the surface), we expect reduced pressure for carbon concentrating mechanisms to supply inorganic carbon to the cell and consider this assumption to be largely reflective of inorganic carbon utilization in the AMZ. Furthermore, model Condition 3 demonstrated that when including a significant utilization of HCO_3 in OMZ autotrophy, neither anammox nor cyanobacteria fractionation can reproduce the isotopic composition of the measured POC (see Supplementary Figs. 6 and 7). However, model Conditions 1, 2, and 4 explore varying degrees of HCO_3 utilization in the surface ocean showing no change in the estimated microbial contribution (see Supplementary Figs. 6 and 7). These exercises support our broad interpretation that CO_2 utilization is most likely involved in OMZ autotrophy, while a mixture of inorganic carbon sources may be utilized in the surface ocean.

- **Carbon Isotope Calculations**

Parameters:

$$r_e = \frac{\Phi_{ezR}}{\Phi_{ezP}} \; ; \; r_o = \frac{\Phi_{amzR}}{\Phi_{amzP}}$$

$$f_{amz} = \frac{\Phi_{amzP}}{\Phi_{amzP} + \Phi_{ezP}}$$

$$f_{onet} = \frac{\Phi_{amzP} - \Phi_{amzR}}{\Phi_{amzP} - \Phi_{amzR} + \Phi_{ezP} - \Phi_{ezR}}$$

Fluxes:

$$\Phi_{net} = \Phi_{ezS} + \Phi_{amzP} - \Phi_{amzR}$$

$$\Phi_{ezS} = \frac{(1 - r_e)(1 - f_{amz})}{f_{amz}(r_e - r_o) + 1 - r_e} * \Phi_{net}$$

$$\Phi_{ezP} = \frac{(1 - f_{amz})}{f_{amz}(r_e - r_o) + 1 - r_e} * \Phi_{net}$$

$$\Phi_{ezR} = r_e \Phi_{ezP}$$

$$\Phi_{amzP} = \frac{f_{amzP}}{f_{amz}(r_e - r_o) + 1 - r_e} * \Phi_{net}$$

$$\Phi_{amzR} = r_o \Phi_{amzP}$$

Carbon Pools and Microbial Contribution:

$$\delta_{OCez} = \delta_{DICez} + \epsilon_{Fez} - r_e \epsilon_{Rez}$$

$$\delta_{OCamz} = \frac{\left[(f_{amz} - 1)(r_e - 1)\delta_{DICez} + (r_e - 1)(r_e \epsilon_{Rez} - \epsilon_{Fez}) + f_{amz}\left(\delta_{DICamz} + \epsilon_{Famz} - r_o \epsilon_{Ramz} + (r_e - 1)(\epsilon_{Fez} - r_e \epsilon_{Rez})\right)\right]}{1 + (f_{amz} - 1) r_e}$$

$$f_{amz} = \frac{(r_e - 1)(\delta_{OCamz} + r_e \epsilon_{Rez} - \delta_{DICez} - \epsilon_{Fez})}{(1 - r_e)\delta_{DICez} + r_e(\delta_{OCamz} - \epsilon_{Fez} + (r_e - 1)\epsilon_{Rez}) + \epsilon_{Fez} + r_o \epsilon_{Ramz} - \delta_{DICamz} - \epsilon_{Famz}}$$

$$f_{onet} = \frac{(r_o - 1)(\delta_{OCez} - \delta_{OCamz})}{(r_o - 1)\delta_{DICez} + \delta_{DICamz} - \epsilon_{Fez} + r_e \epsilon_{Rez} + \epsilon_{Famz} - r_o(\delta_{OCamz} - \epsilon_{Fez} + r_e \epsilon_{Rez} + \epsilon_{Ramz})}$$

Key: Euphotic zone (*ez*) and Anoxic Marine Zone (AMZ); Φ = mass fluxes (*P*-primary productivity, *R*-respiration); r_e (remineralization in euphotic zone) and r_o (remineralization in oxygen minimum zone); δ = isotopic composition of carbon pool, ε = fractionation factor (F-carbon fixation, R-respiration); Organic carbon (*OC*) and dissolved inorganic carbon (*DIC*).
